# Reconfigurable Mechanical Anisotropy in Self‐Assembled Magnetic Superstructures

**DOI:** 10.1002/advs.202002683

**Published:** 2021-02-15

**Authors:** Verner Håkonsen, Gurvinder Singh, José A. De Toro, Peter S. Normile, Erik Wahlström, Jianying He, Zhiliang Zhang

**Affiliations:** ^1^ NTNU Nanomechanical Lab Department of Structural Engineering Norwegian University of Science and Technology (NTNU) Trondheim 7491 Norway; ^2^ School of Biomedical Engineering University of Sydney Sydney NSW 2008 Australia; ^3^ Sydney Nano Institute University of Sydney Sydney NSW 2008 Australia; ^4^ Instituto Regional de Investigación Científica Aplicada (IRICA) and Departamento de Física Aplicada Universidad de Castilla‐La Mancha Ciudad Real 13071 Spain; ^5^ Center for Quantum Spintronics Department of Physics Norwegian University of Science and Technology (NTNU) Trondheim 7491 Norway

**Keywords:** magnetic nanoparticles, mechanical anisotropy, reconfigurability, self‐assembly, super‐magnetostriction

## Abstract

Enhancement of mechanical properties in self‐assembled superstructures of magnetic nanoparticles is a new emerging aspect of their remarkable collective behavior. However, how magnetic interactions modulate mechanical properties is, to date, not fully understood. Through a comprehensive Monte Carlo investigation, this study demonstrates how the mechanical properties of self‐assembled magnetic nanocubes can be controlled intrinsically by the nanoparticle magnetocrystalline anisotropy (MA), as well as by the superstructure shape anisotropy, without any need for changes in structural design (i.e., nanoparticle size, shape, and packing arrangement). A low MA‐to‐dipolar energy ratio, as found in iron oxide and permalloy systems, favors isotropic mechanical superstructure stabilization, whereas a high ratio yields magnetically blocked nanoparticle macrospins which can give rise to metastable *superferromagnetism*, as expected in cobalt ferrite simple cubic supercrystals. Such full parallel alignment of the particle moments is shown to induce mechanical anisotropy, where the superior high‐strength axis can be remotely reconfigured by means of an applied magnetic field. The new concepts developed here pave the way for the experimental realization of smart magneto‐micromechanical systems (based, e.g., on the permanent super‐magnetostriction effect illustrated here) and inspire new design rules for applied functional materials.

## Graphical abstract

The emergence and control of mechanical anisotropy from magnetic anisotropy is demonstrated in self‐assembled magnetic superstructures of nanocubes. Hard magnetic nanoparticles can yield metastable superferromagnetic alignment which gives rise to reversible reconfigurability of the strong mechanical axis by means of an applied magnetic field. The discovery of the “super‐magnetostriction” effect further strengthens the multifunctional aspect of these attractive materials.

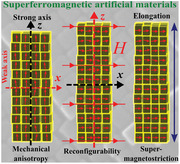

Self‐assembly of magnetic nanoparticles of different sizes and shapes has been successfully applied to design superstructured materials of novel morphologies, including helices, rods, stripes, supercubes, and labyrinths.^[^ ^1^, ^2^, ^3^, ^4^ ^]^ Such magnetic superstructures have gained widespread interest owing to the extraordinary collective properties of the nanoscale building blocks.^[^ ^5^, ^6^, ^7^, ^8^, ^9^ ^]^ In recent years, the focus has shifted from exploring collective magnetic properties to unveiling the mechanical properties of self‐assembled magnetic superstructures, where studies on magnetic nanoparticle‐surfactant nanocomposites have demonstrated exceptionally strong materials.^[^ ^10^, ^11^, ^12^ ^]^ However, the fundamental understanding as to how nanoscale magnetic interactions influence mechanical properties in such structures is yet to be established. Very recently, we showed (for the first time) how nanoscale magnetic interactions can mechanically stabilize self‐assembled magnetic nanocomposite superstructures (based on oleic acid‐coated superparamagnetic magnetite nanocubes), leading to a significant enhancement of mechanical properties (increase in the cohesive energy per nanoparticle up to 45%).^[^ ^13^ ^]^ To date, there is no rigorous fundamental understanding of how intrinsic magnetic properties of materials (e.g., magnetic anisotropy and coercivity) will affect the mechanical properties of superstructures. To maintain stable collective properties over time, it is imperative to develop new materials design rules on how the mechanical properties may be optimized. This knowledge is key to further advance the field of self‐assembled magnetic materials to realize their full potential in practical applications such as sensing devices,^[^ ^14^ ^]^ storage media,^[^ ^15^ ^]^ permanent magnets,^[^ ^16^ ^]^ biomedical applications,^[^ ^2^, ^8^ ^]^ and nanoelectronics.^[^ ^11^, ^17^ ^]^

Here, we use the Monte Carlo (MC) approach to simulate the interaction of macrospins in experimentally obtainable self‐assembled magnetic superstructures (cubic superlattices) based on two different material systems: nanocomposites of oleic acid‐coated 12 nm nanocubes (NCs) of iron oxide (Fe_3_O_4_) and cobalt ferrite (CoFe_2_O_4_). These two magnetic systems exhibit very similar properties in terms of both nonmagnetic interparticle interactions (i.e., van der Waals and steric) and magnetic dipolar interactions,^[^ ^18^, ^19^, ^20^, ^21^ ^]^ as the NCs have similar magnetic moments (as they are of same size and have similar bulk saturation magnetization values: 4.8 × 10^5^ A m^–1^ for iron oxide^[^ ^20^ ^]^ and 4.2 × 10^5^ A m^–1^ for cobalt ferrite^[^ ^21^ ^]^). However, their magnetic anisotropy is notably different, both in magnitude and orientation with respect to their cubic unit cell.^[^ ^22^, ^23^ ^]^ Through a rigorous theoretical investigation of these two excellent model systems, we establish the remarkable relationship between nanoparticle magnetic anisotropy (a local property) and the overall mechanical stability of self‐assembled superstructures, and show how magnetic anisotropy may lead to mechanical anisotropy (i.e., different mechanical properties along different (super)crystallographic directions) through the mediation of magnetostatic interactions. The strength and symmetry of the magnetocrystalline anisotropy (MA) will dictate whether the macrospins relax or remain aligned in an ordered pattern after the in‐field self‐assembly process, strongly influencing the mechanical stability of the system. In particular, a large nanoparticle MA‐to‐dipolar energy ratio may lead to *metastable* states that favor superferromagnetic (SFM) alignment (not to be confused with the dipolar‐driven SFM *ground state* predicted for face‐centered cubic lattices of isotropic macrospins by Luttinger and Tisza^[^ ^24^ ^]^), which in turn results in an anisotropic mechanical stabilization showing an outperforming mechanical stability along the axis of alignment. Such fully aligned (in zero‐field) magnetic materials would behave similarly to natural anisotropic composites, such as wood, which exhibits strong uniaxially aligned microfibers.^[^ ^25^ ^]^ Moreover, we show that this remanent SFM configuration can be reversibly reoriented by a magnetic field applied after self‐assembly, which lays down new rules to design next‐generation bioinspired artificial magnetic materials with field‐controlled reconfigurable mechanical properties. As a remarkable consequence, such SFM superstructures exhibit superior *permanent* “super‐magnetostriction,” a linear magnetostriction one order of magnitude higher than that displayed by the best conventional materials.

To experimentally realize simple cubic superlattices, we synthesize 12 nm monodisperse iron oxide and cobalt ferrite NCs (Figure S1, Supporting Information) by thermal decomposition of a metal‐oleate precursor. The magnetic easy axes of iron oxide (soft magnetic system) and cobalt ferrite (hard magnetic system) lie along the 〈111〉 and 〈100〉 crystallographic directions of the cubic unit cell, respectively, as shown along with transmission electron microscopy (TEM) images of single NCs in **Figure** **1**a. As the cubic nanoparticles are single crystalline, such anisotropy translates directly from the unit cell to the NC. Moreover, the cubic shape of the particles enables the study of superstructures with simple cubic lattices, with NC packing factors reaching up to 1, thus providing as strong interactions as possible between the NCs. Magnetic field‐induced self‐assembly of NCs at the liquid–air interface is used. The oleic acid‐coated NCs are dispersed in hexane and added onto the surface of an immiscible viscous liquid (diethylene glycol). The self‐assembly is performed in an applied vertical magnetic field (electromagnetic setup), in a closed environment where slow evaporation of hexane facilitates the formation of ordered superstructures at the liquid–air interface, as illustrated in Figure 1b (more experimental details can be found in the Experimental Section, Supporting Information).

**Figure 1:**
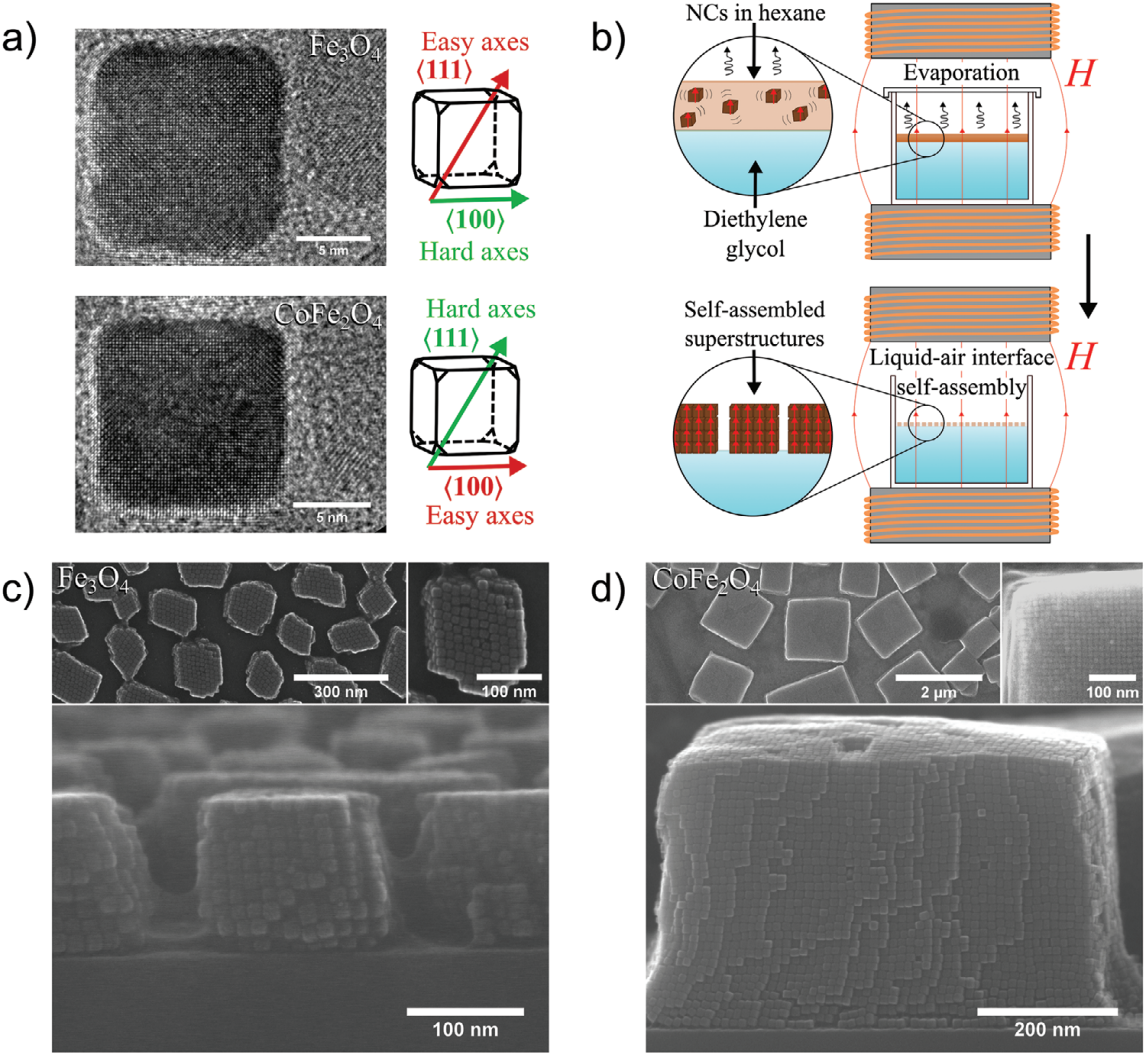
Self‐assembly of ordered iron oxide and cobalt ferrite superstructures. a) TEM images of iron oxide and cobalt ferrite NC building blocks with illustrations of their respective magnetic easy and hard axes along the cube crystallographic directions. b) Liquid–air interface self‐assembly of ferrite NCs in a vertical magnetic field (***H***) generated by an electromagnet. A dispersion of NCs in hexane is added onto the surface of diethylene glycol, where a slow (hexane) evaporation in a closed environment facilitates the in‐field self‐assembly of ordered superstructures. c,d) SEM images (two topmost images in top view and images below in cross‐sectional view) of simple cubic iron oxide and cobalt ferrite supercrystals, respectively. The cobalt ferrite NCs tend to form more well‐defined supercubic shapes, although a coherent stacking of vertical layers can be seen in both cases.

Scanning electron microscopy (SEM) images (Figure 1c,d) reveal the formation of coherently stacked superstructures of both types of NCs in a simple cubic superlattice (see SEM images in Figures S2 and S3, Supporting Information). These observations are consistent with supercrystal structures observed in previous studies of both material systems.^[^ ^2^, ^4^ ^]^ We note that a slight deviation from the perfect simple cubic superlattice is observed in the case of iron oxide (Figure 1c and Figure S2, Supporting Information), presumably arising from the significantly smaller size of these superstructures, compared with the cobalt ferrite supercrystals. A smaller size makes the superstructures less mechanically stable after self‐assembly, as a result of the super‐size effect thoroughly discussed in our previous work,^[^ ^13^ ^]^ and therefore more prone to distortions during the subsequent lift‐off step (these distortions are less evident for larger structures, but can nevertheless be clearly seen in some cobalt ferrite superstructures, e.g., in Figure S3e, Supporting Information). Moreover, a stronger in‐plane dipolar repulsion between vertical macrospins in the iron oxide simple cubic superstructures (owing to a higher NC moment than in cobalt ferrite) during in‐field self‐assembly may cause additional distortions. Considering the previously confirmed simple cubic superlattice^[^ ^2^ ^]^ and the fact that higher quality supercrystals definitely are present on our samples (see Figure S2, Supporting Information), we stress that this particular crystal structure is a valid approximation for the iron oxide NC assemblies. We also observe other distinct 3D iron oxide NC arrangements, such as structures with the NCs aligned with their 〈111〉‐directions along the vertical axis (exhibiting in‐plane hexagonal symmetry, as thoroughly discussed in a recent work^[^ ^13^ ^]^), as well as quasi‐hexagonal superstructures (Figure S4, Supporting Information). The absence of any such noncubic superstructures in the cobalt ferrite NC assemblies corroborates the 〈100〉‐direction of the anisotropy in these particles. To develop a conceptual understanding of the effect of magnetic anisotropy on mechanical properties, we compare the two material systems on equal grounds by only considering the more stable simple cubic arrangement for iron oxide as based on previous reports.^[^ ^13^, ^26^ ^]^

During self‐assembly, the macrospins of the NCs in a given solution will align in the direction of the (sufficiently strong) applied magnetic field and remain aligned after the assembly process until the field is switched off. To gain deeper insight into how the NC macrospins interact in the model systems after self‐assembly, we use an MC method based on a model reported in a previous study (Metropolis‐Hastings algorithm in the NVT ensemble).^[^ ^13^ ^]^ This model includes the following interparticle interactions: i) van der Waals interaction between NC cores, ii) steric repulsion resulting from overlapping adsorbed oleic acid chains (note that this steric model does not account for any possible resistance to shear motion), and iii) magnetic dipole–dipole interactions. The potentials for the van der Waals and steric interactions are identical for both material systems (however, the strength of these interactions may vary depending on interparticle spacing, which again depends on magnetic configuration). The sum of these three terms is defined as the *cohesive energy*, whose sign is positive by convention. In addition, the MA is included as an intraparticle potential, favoring macrospin alignment along the magnetic easy axes (with MA constants of −1.3 × 10^4^ J m^–3^ for iron oxide^[^ ^22^ ^]^ and 2.6 × 10^5^ J m^–3^ for cobalt ferrite^[^ ^23^ ^]^), which may indirectly affect the dipolar interaction, and thus the cohesive energy. Furthermore, we consider a translationally dynamic system of NCs, in which fluctuations in the NC positions contribute to a more physically accurate system than the previously used static system.^[^ ^13^ ^]^ In this model, we build magnetic superstructures with square cross sections of *n* × *n* cubes (*n* ranging from 2 to 6), with *h* layers stacked heightwise (*h* ranging from 1 to 16); the aspect ratio, *A*, of a superstructure being *A* = *h*/*n*. In the initial state, a saturating vertical magnetic field (as that used during the self‐assembly experiments) has just been switched off and all macrospins are aligned vertically along the *z*‐axis. Thereafter, we perform an initial rotational macrospin relaxation of 10^4^ MC iterations/steps, followed by 5 × 10^5^ steps (both rotationally for macrospins and translationally for NC positions) at room temperature (RT, 298 K) to obtain extensive statistical data of the superstructures after reaching thermal equilibrium. More details regarding the MC simulations are available in the Supporting Information.

The way the macrospins relax and interact after removal of the external magnetic field is shown in **Figure** **2**a,b for iron oxide and cobalt ferrite systems of different *A*. A clear distinction can be made between the two different material systems in thermal equilibrium. In the iron oxide system (Figure 2a) of relatively weak 〈111〉‐symmetry MA, the macrospins (red arrows) quickly relax from the vertically aligned state, reducing both the MA and magnetostatic energies (intrawell relaxation). These spins continue to fluctuate through the interwell Néel relaxation mechanism, i.e., superparamagnetic fluctuations.^[^ ^27^ ^]^ This is consistent with previous findings in identical (but translationally static) systems.^[^ ^13^ ^]^ In the cobalt ferrite case, however, the macrospins do not undergo any of the above relaxation processes and remain vertically aligned owing to the strong MA, and, crucially, to the fact that their initial magnetization already lies along one of the easy axes of the NCs. Hence, the macrospins are “blocked” in one of the intraparticle MA potential wells, consistent with experimental magnetic measurements in the literature of cobalt ferrite NCs of similar size.^[^ ^28^ ^]^ Therefore, the cobalt ferrite superstructure systems self‐assembled in a vertical magnetic field exhibit MA‐induced “SFM” (full parallel alignment of macrospins) at RT after the applied field is switched off.

**Figure 2:**
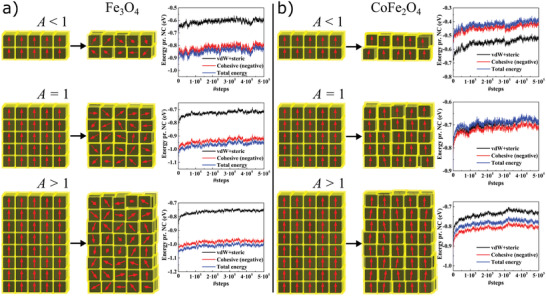
Spin relaxation and interaction energies in in‐field self‐assembled iron oxide and cobalt ferrite superstructures of different aspect ratios (*A* = {2/5, 5/5, 8/5}). a) The macrospins (red arrows) in the iron oxide systems relax and interact as a dynamic system of fluctuating spins, leading to a significant increase in cohesive energy per NC relative to the nonmagnetic system (i.e., van der Waals + steric energies) for all the considered aspect ratios. Overall, these superparamagnetic superstructures are mechanically stabilized by attractive magnetic interactions. b) The blocked macrospins remain vertically aligned in the initial state in the cobalt ferrite systems owing to the strong MA favoring the 〈100〉‐directions, giving rise to MA‐induced “SFM.” The cohesive energy per NC increases for *A* > 1 as a result of dominating attractive magnetic dipolar interactions (shape anisotropy parallel to magnetization), and decreases for *A* < 1 owing to dominating repulsive magnetic dipolar interactions (shape anisotropy perpendicular to magnetization). Dark and yellow cubic boxes in the snapshots are the NC core and oleic acid surfactant layer, respectively.

Based on the above observations, we next establish the effect of MA on the overall mechanical stability. The plots in Figure 2 show energies per NC (i.e., van der Waals + steric, cohesive, and total energy) as a function of the number of MC steps (the initial fast intrawell rotational relaxation is not displayed and can be found in Figure S5, Supporting information). Here, van der Waals + steric energy is basically the nonmagnetic cohesive energy, whereas the (total) cohesive energy also includes the magnetic dipolar interactions (displayed with negative values for comparison with other energies). The total energy also includes the intraparticle MA energy, a negative contribution for iron oxide systems and positive for cobalt ferrite (from the sign of the corresponding anisotropy constants). Regardless of aspect ratio (i.e., whether *A* < 1, *A* = 1, or *A* > 1), the iron oxide systems (Figure 2a) show a significant increase in cohesive energy per NC owing to attractive interactions between relaxed macrospins. For the high MA cobalt ferrite systems (Figure 2b), we observe a different scenario. Here, the aspect ratio, and thus the shape anisotropy, of the superstructure dictates whether the introduction of vertically aligned macrospins is beneficial for the overall cohesion. As expected, vertically aligned macrospins enhance the mechanical stability only for high aspect ratio superstructures (*A* > 1), i.e., with shape anisotropy along the initial magnetization direction, because of the majority of favorable head‐to‐tail spins. Conversely, a structure with *A* < 1 yields a net magnetic dipolar repulsion, and thus a destabilization, owing to predominant parallel side‐by‐side macrospins. For *A* = 1, the cohesive energy per NC remains virtually unchanged after taking the dipolar interaction into account, as the energy cost of having spins configured in parallel side‐by‐side is perfectly balanced by the benefit of head‐to‐tail interactions between vertically stacked layers. The energy fluctuations observed in the RT‐blocked cobalt ferrite systems arise mainly from translational fluctuations in the NC positions.

When the systems reach thermal equilibrium, the cohesive energy as a function of MC steps flattens out and reaches a plateau about which it fluctuates owing to thermal excitations and relaxations (see plots in Figure 2). To gain deeper insight into the mechanical stability of the two studied magnetic superstructure systems, we analyze the mean cohesive energy of the plateau as a function of superstructure (reciprocal) *A*, as displayed in **Figure** **3**a,b. In general, the mean cohesive energy per NC increases as the size of the superstructure increases (with decreasing standard deviation owing to a reduction in the degree of thermal fluctuations). However, a distinct difference can again be seen between the two material systems. The iron oxide systems (Figure 3a) follow a linear trend for structures of the same cross section (*n*‐number), an observation consistent with our previous study on translationally static systems.^[^ ^13^ ^]^ Data points in Figure 3a corresponding to the same *n*‐number fit well to the linear function *E*_coh_ = *a*(1/*A*) + *b* (details of each individual fit can be found in Figure S6a, Supporting Information), for which the parameters *a* and *b* also follow a linear relationship with respect to 1/*n* (*a* = *a*_1_(1/*n*) + *a*_2_, and *b* = *b*_1_(1/*n*) + *b*_2_), as displayed in the inset of Figure 3a. The equation for the cohesive energy per NC of a translationally dynamic iron oxide system of any size (provided a cross section of size *n* ≥ 2) can therefore be written as follows

(1) $$E_{\mathrm{coh}}=\frac{a_{1}}{h}\;+\frac{b_{1}}{n}+E_{\mathrm{bulk}}$$

in which *E*_bulk_ = *b*_2_, as established in a previous report (valid for mechanically isotropic superstructures).^[^ ^13^ ^]^ Interestingly, the cobalt ferrite systems (Figure 3b) appear to follow an exponential trend (*E*_coh_ = *b*exp[*a*(1/*A*)]) for the data points corresponding to the same *n*‐number (details of each individual fit can be found in Figure S6b, Supporting Information). This exponential trend becomes more obvious as the cross section becomes larger and can be understood from the vertically aligned spins initially repelling each other in low *A* structures (*A *< 1) and starting to overall attract each other as *A* > 1 (Figure 2b). This leads to the observed exponential decrease in cohesive energy per NC with 1/*A* (semi‐log plots of the data in Figure 3b support an exponential relationship; see Figure S7, Supporting Information). Also, in this case, the fitting parameters, *a* and *b*, follow a linear relationship when plotted against 1/*n* (shown in the inset of Figure 3b). A summary of the obtained fitting parameters is given in **Table** **1** for both material systems (note that the *a*‐parameters of the two systems have different units). The observation of *a*_2_ ≈ 0 for both systems supports that the slope of the curve fitted to the different *n*‐numbers in Figure 3a,b converges toward 0 as *n* → ∞, which can also be geometrically inferred from these plots. Given the linearity in the exponential fitting parameters (with *a*_2_ = 0), the cohesive energy per NC for any cobalt ferrite system size (provided a square cross section of size *n* ≥ 2) can be written as follows

(2) $$E_{\mathrm{coh}}=\left(\frac{b_{1}}{n}+E_{\mathrm{bulk}}\right)\;\exp\left(\frac{a_{1}}{h}\right)$$

where *E*_bulk_ = *b*_2_ (see the Supporting Information for details regarding the derivation). Following a Taylor expansion at large *A* (i.e., taken at 1/*A* = 0), Equation (2) can be approximated as

(3) $$E_{\mathrm{coh}}\approx \frac{a_{1}E_{\mathrm{bulk}}}{h}+\frac{b_{1}}{n}+E_{\mathrm{bulk}}$$

and adopts the same form as Equation (1) (details in the Supporting Information). We expect the expression in Equation (2) to hold also for magnetic materials different from cobalt ferrite, given that the macrospins are blocked and parallelly aligned.

**Figure 3:**
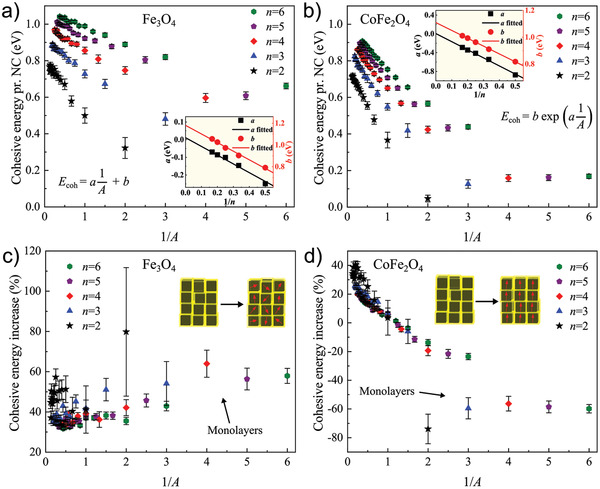
RT equilibrium cohesive energy per NC and the %increase in cohesive energy owing to magnetic interactions, as a function of superstructure (reciprocal) aspect ratio. The cohesive energy shows a linear relationship for a) iron oxide and an exponential relationship for b) cobalt ferrite, when considering superstructures of the same number of NCs in the cross section (*n*‐number). The insets show the *a* and *b* parameters for both material systems, obtained for data points corresponding to the same *n*‐number (see fitting details in Figure S6, Supporting Information) and plotted as a function of 1/*n*, which in both cases reveal linear relationships (see obtained fitting parameters in Table 1). c) All the iron oxide superstructures show a magnetically induced mechanical stabilization which increases with decreasing system size (both *n* and *h*). d) The %increase in cohesive energy shows significant negative values for cobalt ferrite monolayers, indicating an in‐plane mechanical destabilization, which increases and becomes positive (for *A* > 1) as layers are stacked in the vertical direction, thereby stabilizing the structure overall. An isotropic mechanical stabilization is observed in the iron oxide systems, whereas anisotropy in the mechanical cohesion is observed for the cobalt ferrite systems. The cohesive energy data are presented as mean ± standard deviation (sample size 2000). Details on linear regression and exponential curve fitting are given in the Experimental Section (Supporting Information). The %increase in cohesive energy is calculated relative to mean values of the nonmagnetic systems.

**Table 1:** Decomposition of the parameters *a* and *b* (obtained from the curve fitting in Figure 3a,b) into parameters *a*_1_, *a*_2_, *b*_1_, and *b*_2_, for the iron oxide and cobalt ferrite superstructure systems

Material system^a)^	*a* = *a*_1_(1/*n*) + *a*_2_			*b* = *b*_1_(1/*n*) + *b*_2_		
	*a*_1_	*a*_2_	*R* ^2^	*b*_1_ [eV]	*b*_2_ [eV]	*R* ^2^
Fe_3_O_4_	−0.53(4)	0.02(1)	0.985	−0.81(5)	1.19(1)	0.991
CoFe_2_O_4_	−1.6(1)	−0.02(2)	0.986	−0.60(2)	1.112(4)	0.998

^a)^ The parameters *a*_1_ and *a*_2_ have units of eV for the iron oxide systems, but are dimensionless for the cobalt ferrite systems. Each value in parentheses is a fitting error and refers to the least significant digit of the corresponding parameter. The goodness‐of‐fit is expressed through the *R* ^2^ value. The high relative errors on the *a*_2_ values are, naturally, associated with high significance probability values (*p* = 0.14 and *p* = 0.57 for the iron oxide and cobalt ferrite systems, respectively). *p* > 0.05 implies that the null hypothesis of a zero intercept (i.e., *a*_2_ is equal to zero) cannot be rejected, i.e., we conclude *a*_2_ = 0 for each system.

The comparison of the two material systems in Figure 3a,b shows that an iron oxide system is generally more stable than a cobalt ferrite system of the same size, although there are more fluctuations in the data points for iron oxide arising from fluctuations in both macrospin orientation and NC positions (as opposed to cobalt ferrite where the spins are effectively locked in the positive *z*‐direction). Even for high *A*, the minimization of the demagnetizing field in the case of the relaxed iron oxide macrospins results in a more energetically favorable state than the net attractive interactions in cobalt ferrite systems with macrospins locked along the vertical easy axes. The influence of magnetic anisotropy is well expressed in the mechanical stability of the cobalt ferrite systems in Figure 3b, through the rapid increase in cohesive energy per NC as vertical layers are stacked on top of each other, especially for the largest considered systems of this study (with *n* = 6). Moreover, in both material systems, a super‐size effect is evident, implying on average a mechanical destabilization as the superstructure systems become smaller in size, consistent with our previous study.^[^ ^13^ ^]^

Further analysis of the variation in cohesive energy after taking magnetism into account in the two different systems reveals the influence of magnetic anisotropy on the mechanical stability. The percentage increase (%increase) in cohesive energy is calculated relative to the equivalent nonmagnetic system of iron oxide and cobalt ferrite (i.e., where the simulation is rerun after magnetism has been removed (details in Figure S8, Supporting Information), enabling a comparison of the two material systems on equal footings) and plotted in Figure 3c,d, respectively. The iron oxide systems are consistently stabilized regardless of size, reaching at least an enhancement of 29% (bulk system mean value) and up to 80% for the smallest monolayer (*n* = 2). Compared with an enhancement in mechanical stability by up to 45% for the translationally static systems,^[^ ^13^ ^]^ the value of 80% reflects the higher level of importance of magnetism in real NC fluctuating systems. Generally, the iron oxide monolayers and the superstructures of smallest cross section (*n* = 2) benefit more from the magnetic character of the NCs and thus show a higher %increase in cohesive energy than the systems of larger size (this is a direct consequence of the shorter ranged magnetic dipole–dipole interactions, 1/*r* ^3^, compared with the van der Waals interactions, 1/*r* ^2^). For the cobalt ferrite systems, however, we observe a very different trend. Here, the monolayers are destabilized by the introduction of magnetism (negative %increase in cohesive energy of around −60%), as the macrospins are blocked by MA perpendicular to the in‐plane shape anisotropy axes. As layers are stacked on top of each other, this effect weakens and the %increase in cohesive energy eventually becomes positive overall (i.e., for *A* > 1) owing to the strong attraction between vertical macrospins, as anticipated by Figure 2. Hence, as a direct consequence of magnetic anisotropy, a superstructure of this kind will be mechanically destabilized in‐plane and stabilized in the vertical direction (which is also reflected in the difference in interparticle spacings along the Cartesian axes, shown in Table S1, Supporting Information). Interestingly, beyond the monolayer regime, the %increase as a function of 1/*A* in Figure 3d seems to fall onto the same (master) curve regardless of superstructure size, corroborating the idea that shape anisotropy (determined by aspect ratio) governs the contribution of magnetism to the cohesive energy in these ordered systems of blocked macrospins.

It is clear that a low MA‐to‐dipolar energy ratio, as found in the iron oxide system, implies an isotropic mechanical stabilization of the superstructure. Remarkably, in the interesting case of near‐zero MA, shape anisotropy could enforce a collective super‐antiferromagnetic alignment, yielding an even stronger isotropic mechanical stabilization (note that this configuration is driven solely by dipolar interactions, in contrast with the SFM state described above). This is observed in high *A* superstructures of iron oxide in which the MA has been set to zero. A candidate for a real system with near‐zero MA is permalloy (Ni_80_Fe_20_),^[^ ^29^ ^]^ where we can expect such super‐antiferromagnetic alignment at RT. See the Supporting Information (discussion and Figures S9–S14, Supporting Information) for a thorough treatment of the collective magnetism and mechanical stabilization in near‐zero MA superstructure systems. Given similar dipole–dipole interactions, our results demonstrate that the relative mechanical stability of the three different types of macrospin configurations (resulting from different MA‐to‐dipolar energy ratios) considered in this work follows the trend: SFM < disordered < super‐antiferromagnetic. The reader is referred to the Supporting Information for a more comprehensive discussion (accompanying text and Figures S15 and S16, Supporting Information) concerning the influence of macrospin configuration on the mechanical stabilization of superstructures.

We have shown in this study that a magnetic superstructure in thermal equilibrium with SFM ordering (e.g., cobalt ferrite NCs self‐assembled in‐field) is *overall* less mechanically stable than an equivalent disordered system (e.g., superparamagnetic iron oxide). However, if we calculate the cohesive energy solely along the magnetization direction (*z*‐axis), we obtain an outperforming magnetic enhancement of mechanical stability for cobalt ferrite. We illustrate this in **Figure** **4**a, where a cobalt ferrite superstructure without shape anisotropy (i.e., in this context meaning a perfect cubic shape, with *n* = *h* = 6, *A* = 1), which on average should show no significant enhancement in mechanical stability, displays a magnetic increase in cohesive energy of ≈90% along the *z*‐axis. Conversely, along the *x*‐axis, a destabilization of −40% is observed (with an expectedly similar value of −42% along the *y*‐axis). In fact, this (shape) isotropic superstructure is 4 times stronger in the *z*‐direction than in the *x*‐direction, an effect which will be enhanced as the aspect ratio increases (i.e., *A* > 1). In comparison, the equivalent superparamagnetic iron oxide superstructure shows an increase in cohesive energy of ≈55% along all three Cartesian axes (Figure S17, Supporting Information), as indeed would be expected for a mechanically isotropic system. In a superstructure of vertically aligned macrospins, it is the absence of short‐ranged exchange interactions, responsible for energetically favorable parallel spin alignment in traditional ferromagnetic systems, which yields a significant in‐plane destabilization effect for the macrospins. Therefore, such MA‐induced SFM macrospin alignment will lead to mechanical anisotropy, which is well expressed through the axis‐dependent enhancement/suppression of mechanical stability in Figure 4a. As a prerequisite for the emergence of mechanical anisotropy in such magnetically saturated configurations, the MA energy barrier between easy directions (〈100〉 in our case) should be strong enough to block the macrospins against thermally induced flipping, as well as to prevent a reorientation driven by strong dipolar interactions between neighboring nanoparticles. This will stabilize the vertically aligned macrospin configuration in a metastable state (i.e., as the super‐antiferromagnetic alignment (Figure S15, Supporting Information) yields the ground state, where the MA energy is minimized equivalently to the metastable SFM alignment). We have already realized the self‐assembly of superstructures (Figure 1) which are expected to show this type of anisotropic mechanical behaviour predicted by our MC simulations. Proving this emergence of mechanical anisotropy experimentally will provide new opportunities to design multifunctional materials for new potential applications.

**Figure 4:**
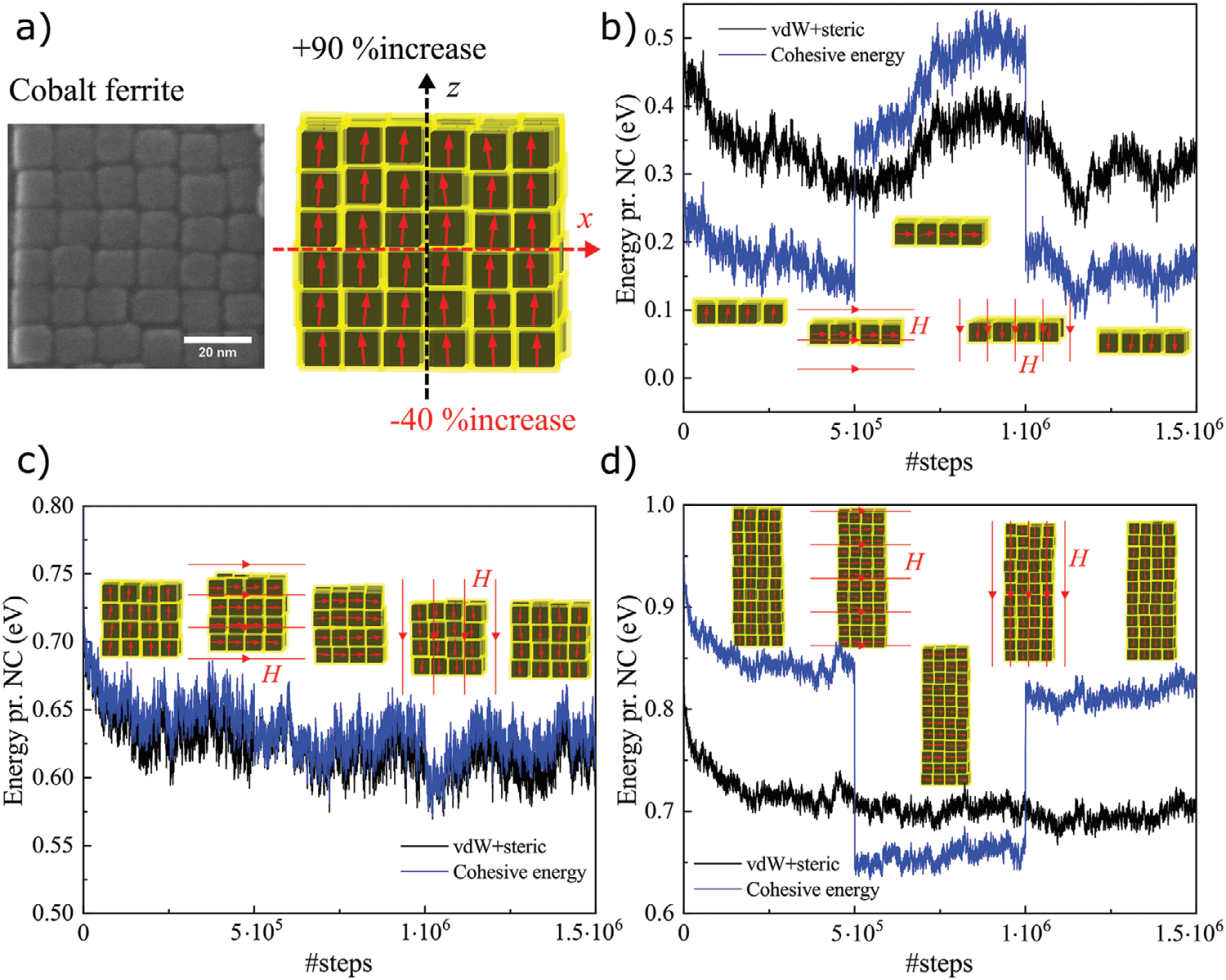
Mechanical anisotropy and reconfigurability of mechanical properties via applied magnetic fields. a) A “shape isotropic” cobalt ferrite superstructure (high‐resolution SEM image on the left) with vertically aligned macrospins displays mechanical anisotropy, resulting in axis‐dependent enhancement in the mechanical stability. Owing to this SFM alignment, a %increase in cohesive energy of +90% and −40% along the vertical and horizontal axis, respectively, is obtained. b–d) After 5 × 10^5^ MC steps, the initial vertically aligned macrospin state of three cobalt ferrite superstructures in thermal equilibrium is switched to a horizontal SFM configuration by means of an applied magnetic field (1 T during 1000 steps). After switching to the horizontal configuration: b) the monolayer (*A* < 1) is observed to undergo a significant mechanical stabilization overall, c) the perfect supercube (*A* = 1) does not display any notable change (except for the obvious horizontal flipping of the high‐strength axis), and d) the pillar (*A* > 1) becomes overall significantly destabilized. When switching back to the vertically aligned state after 10^6^ steps (this time with macrospins pointing downward), the mechanical properties are reversed back to the starting point.

Our results suggest new design rules for applied materials with predefined properties. For example, self‐assembled superstructures comprising nanoparticles with low MA‐to‐dipolar energy ratio (isotropically maximizing cohesive energy) could be exploited in applications requiring long‐term mechanical stability (such as micro‐electromechanical systems (MEMS),^[^ ^30^ ^]^ sensors,^[^ ^31^ ^]^ magnonic crystals,^[^ ^32^ ^]^ or certain scaffolds for tissue engineering^[^ ^33^ ^]^). Superstructures with higher MA‐to‐dipolar ratios (such as the cobalt ferrite systems considered here), which can sustain permanent SFM at RT, are recognized as promising candidates for use in a variety of applications. First and foremost, the full permanent alignment of the macrospins makes these systems very appealing as permanent (micro)magnets, given that the associated perfectly square hysteresis loop optimizes the (*BH*)_max_ energy product (i.e., the product of magnetic flux density and magnetic field).^[^ ^16^ ^]^ Yet, the most exciting aspect of such magnetically blocked assemblies is the possibility of magnetic field control of the mechanical anisotropy and overall cohesion through changes in the permanent magnetization direction. This is illustrated in Figure 4b–d, in which the magnetic field reversibly toggles the total moment of the system between the easy axes of the superstructure. These mechanically anisotropic nanostructures would behave as *smart wood* (Figure S18, Supporting Information), where the high‐strength axis (analogous to the microfibers in wood^[^ ^25^ ^]^) could be externally reconfigured as desired, also identifying them as promising candidates in structural engineering.^[^ ^34^ ^]^ Furthermore, we demonstrate another emerging novel property of such SFM superstructures, namely, enhanced magnetostriction. Such linear “super‐magnetostriction” outperforms that of conventional materials (Terfenol‐D) by at least one order of magnitude,^[^ ^35^, ^36^ ^]^ as illustrated in **Figure** **5**. Moreover, in stark contrast with continuous media, this super‐magnetostriction is permanent, i.e., the enhanced strain remains until a differently oriented field is applied. One may thus envision super‐magnetostrictive microsensors/actuators,^[^ ^36^ ^]^ tailored by the strength of the magnetic interactions as well as by the nature of the surfactants (which may be readily explored to optimize the super‐magnetostrictive effect far beyond the value calculated here: ≈2%), enabling magnetic field‐controlled variation of strain and elastic modulus through the mechanisms simulated here. Note that the super‐magnetostriction effect schematized in Figure 5 is permanent (yet easily reconfigurable) and *discrete*. A continuous and reversible super‐magnetostriction, analogous to conventional linear magnetostriction, will be obtained in the superparamagnetic iron oxide superstructures studied here. These isotropic systems are expected to show a negative strain in the external magnetic field direction, thus the magnetization direction, and a positive strain perpendicular to the field (note, however, that this strain will be less profound than that of the anisotropic discrete case). The field dependence of both strains will be ultimately governed by the Langevin magnetic response of the fluctuating macrospins, whose linear range and sensitivity—with sensor applications in mind—can be tailored simply through the NC magnetic moment.

**Figure 5:**
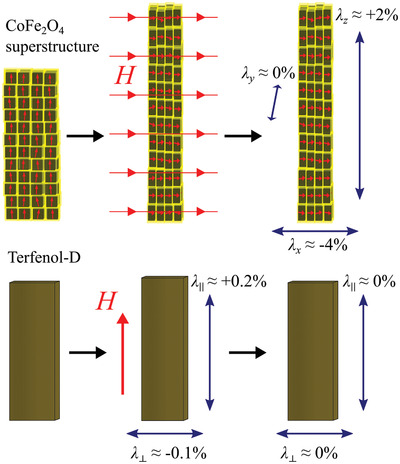
Permanent super‐magnetostriction in cobalt ferrite superstructures. SFM cobalt ferrite superstructures exhibit superior linear magnetostriction (strain induced by the applied magnetic field, *λ*) by one order of magnitude compared with the best conventional magnetostrictive materials. This “super‐magnetostriction” is mediated by dipolar interactions upon macrospin flipping (from vertical to horizontal alignment and vice versa) and is always negative parallel to the applied magnetic field and positive perpendicular to the field. Moreover, there will be a near‐zero strain along the other axis perpendicular to the field, because the configuration of the magnetic moments remains unchanged after spin flipping. The magnetostriction takes on values of +2% and −4% in the vertical and horizontal direction, respectively, of the selected superstructure (*n* = 4, *h* = 12), compared with +0.2% in the saturated field direction for the alloy Terfenol‐D, which exhibits the highest *λ*‐value among conventional materials.^[^ ^35^ ^]^ Moreover, the saturation magnetostriction in such metastable SFM superstructures is permanent (i.e., it remains after field removal), as opposed to continuous materials. The magnetostrictive elongations/compressions in the figure are consistently exaggerated for illustrative purposes.

In summary, our simulations predict that nanoparticle magnetocrystalline anisotropy (both in magnitude and symmetry), together with shape anisotropy, has a profound effect on the mechanical stability of self‐assembled superstructures of nanoparticles. By simply choosing magnetic materials of appropriate MA, the mechanical properties of the superstructures can be magnetically tailored without any change in the structural design (i.e., nanoparticle packing arrangement and density). Low MA‐to‐dipolar energy ratios give rise to isotropic mechanical stabilizations of superstructures either through disordered (superparamagnetic) systems of macrospins (as displayed in an iron oxide system), or through even stronger ordered ground state configurations such as shape anisotropy‐enforced super‐antiferromagnetic alignment (prompting its experimental realization in superstructures of permalloy nanocubes). A high MA‐to‐dipolar ratio leads to fully ordered macrospin states, such as SFM configurations, ultimately resulting in mechanical anisotropy (e.g., in‐plane mechanical destabilization and out‐of‐plane stabilization). The realization of MA‐induced SFM states, as observed in cobalt ferrite superstructures, creates a roadmap to new design principles exploiting anisotropic high‐strength‐axis materials with field‐controlled reconfigurable mechanical properties. The conceptual understanding built in this work paves the way toward the experimental realization, e.g., through a combination of magnetic measurements and mechanical nanoindentation testing, of reconfigurable mechanical anisotropy in ordered superstructures of magnetic nanocubes. In such RT‐blocked systems, notably different mechanical properties (such as elastic modulus, strength and hardness), and thus super‐magnetostrictive responses, are expected for in‐plane and out‐of‐plane measurements.

## Experimental Section

Experimental details are available in the Supporting Information.

## Conflict of Interest

The authors declare no conflict of interest.

## Supporting information

Supporting Information

## Data Availability

The data that support the findings of this study are available from the corresponding author upon reasonable request.
