# Supplementary material for: Reconfigurable Mechanical Anisotropy in Self‐Assembled Magnetic Superstructures
Source: Adv Sci (Weinh). 2021 Feb 15;8(8):2002683. doi: 10.1002/advs.202002683 (PMC8061348; doi:10.1002/advs.202002683)
Supplement: Supplementary file 1 — Supporting Information [file ADVS-8-2002683-s001.pdf]

© 2021 Wiley-VCH GmbH

ADVANCED SCIENCE
Open Access

Supporting Information

for *Adv. Sci.,* DOI: 10.1002/advs.202002683

Reconfigurable Mechanical Anisotropy in Self-Assembled Magnetic Superstructures

Verner Håkonsen, Gurvinder Singh, José A. De Toro, Peter S. Normile, Erik Wahlström, Jianying He, and Zhiliang Zhang**

© Copyright 2021. WILEY-VCH GmbH.

Supporting Information

Reconfigurable Mechanical Anisotropy in Self-Assembled Magnetic Superstructures

Verner Håkonsen, Gurvinder Singh, José A. De Toro, Peter S. Normile, Erik Wahlström, Jianying He, and Zhiliang Zhang**

Experimental Section

Synthesis of Monodisperse Iron Oxide (Fe_3O_4) Nanocubes

Monodisperse iron oxide nanocubes (NCs) were synthesized by thermal decomposition of iron oleate precursor in the presence of sodium oleate and octadecene, according to a protocol previously reported in the literature.[1] Firstly, the iron oleate precursor was synthesized by mixing iron(III) chloride hexahydrate ($FeCl_3·6H_2O$, Sigma Aldrich, ≤99%) (5.39 g; 20 mmol), sodium oleate (TCI, ≤97%) (18.25 g; 60 mmol), deionized water (30 mL), hexane (70 mL) and ethanol (40 mL) in a 250 mL round bottom flask. The reaction mixture was vigorously stirred at 70 °C for 4 h under argon atmosphere. After the reaction, dark red organic product was separated from the aqueous phase and washed three times with deionized water to remove byproducts. The product was further purified in a rotary evaporator to remove residual hexane, ethanol and water, before being transferred to a glass vial and stored in a fridge (4 °C). Iron oxide NCs were synthesized by adding as-prepared iron oleate (1.62 g) and sodium oleate (420 mg) to a 100 mL round bottom flask containing octadecene (Sigma Aldrich, 25 mL). The reaction mixture was magnetically stirred and heated up to 320 °C with a heating rate of 3 °C min^{-1}, and kept at this temperature for 45 min, before being cooled down to room temperature (RT). The NCs were obtained after washing three times with a mixture of toluene and isopropanol to remove oleate residues, and thereafter stored in toluene. The size of the iron oxide NCs was measured to be 12 ± 1 nm (scanning transmission electron micrograph in Figure S1a below).

Synthesis of Monodisperse Cobalt Ferrite ($CoFe_2O_4$) Nanocubes

Monodisperse cobalt ferrite NCs were synthesized by thermal decomposition of iron-cobalt oleate precursor, according to a modified protocol reported in the literature.[2] For preparing the iron-cobalt oleate precursor, the same protocol as for the iron oxide was utilized except for the addition of both iron(III) chloride hexahydrate (3.60 g; 13.33 mmol) and cobalt(II) chloride hexahydrate ($CoCl_2·6H_2O$, Sigma Aldrich, ≤97%) (1.59 g; 6.67 mmol), in a molar ratio of 2:1 to obtain the desired stoichiometry of the cobalt ferrite NCs. Cobalt ferrite NCs were synthesized by adding as-prepared iron-cobalt oleate (1.57 g) and sodium oleate (530 mg) to a 100 mL round bottom flask containing octadecene (25 mL). The reaction mixture was magnetically stirred and heated up to 325 °C with a heating rate of 3 °C min^{-1}, and kept at this temperature for 45 min, before cooling down to RT. The NCs were obtained after washing three times with a mixture of toluene and ethanol to remove oleate residues, and thereafter stored in

toluene. The size of the cobalt ferrite NCs was measured to be 12 ± 1 nm (scanning transmission electron micrograph in Figure S1b below).

Liquid-Air Interface Self-Assembly of Nanocubes in Magnetic Field

Ordered magnetic superstructures were fabricated by liquid air-interface self-assembly of NCs in an electromagnetic setup, according to a previous report in the literature.[3] The NCs were dispersed in hexane, in an excess amount of oleic acid surfactant, and added onto the surface of a liquid subphase of diethylene glycol (2 mL), inside a polyethylene well of diameter and height ≈2 cm. For iron oxide, 40 µL dispersion of NC concentration 9 mg mL^{-1}, and oleic acid concentration 3 µL mL^{-1}, was added. For cobalt ferrite, 40 µL dispersion of NC concentration 6 mg mL^{-1}, and oleic acid concentration 4 µL mL^{-1}, was added. A glass slide was used to cover the well to slow down evaporation of hexane, before the immediate application of a vertical magnetic field (1200 G; gradient 300 G cm^{-1} causing attractive force downwards). The setup was left undisturbed at RT until complete evaporation of hexane (up to 1 h). Subsequently, acetonitrile was injected underneath the self-assembled film by means of a syringe, to raise the superstructures away from the diethylene glycol and ease the transfer onto a silicon substrate through lift-off. The lift-off was followed by drying under vacuum to remove residues of diethylene glycol. Thorough washing of samples with ethanol by means of a squirt bottle was done to remove excess oleic acid, before subsequent characterization.

Characterization

Scanning electron microscopy (SEM) images and scanning transmission electron microscopy (STEM) images were captured in a Hitachi S-5500 S(T)EM, with acceleration voltage 30 kV. A diamond tip scriber was used to cut the silicon substrate in half prior to cross-sectional imaging. Transmission electron microscopy images were captured in a JEOL 2100F, with an acceleration voltage of 200 kV.

Statistical Analysis

All the average cohesive energy data were normalized with respect to the number of nanoparticles in the superstructure, and are reported as means together with standard deviation values. The sample size was 2000 in all cases. Linear regression models were obtained using the `fitlm` function of MATLAB (The MathWorks Inc., Natick, MA, USA), with errorbars on `y`-values (`ey`) being used as weights, i.e., `fitlm(x,y,'weight',1./ey)`. Standard errors on the slope and intercept parameters were extracted as follows:

```
f = fitlm(x,y,'weight',1./ey);
error_slope = f.Coefficients.SE(2);
error_intercept = f.Coefficients.SE(1);
```

For cases in which a large relative error is found for the intercept value, the significance probability value of the intercept, *p*, was also extracted:

```
f.Coefficients.pValue(1);
```

A value of *p* larger than a typical cutoff (e.g., $p > 0.05$, as used here) implies that the Null Hypothesis of the intercept cannot be rejected, i.e., the data indicates that the intercept is likely to be zero. A non-linear least squares method based on a Levenberg-Marquardt algorithm was employed to obtain the data fits to an exponential function. Furthermore, the percentage increase (%increase) in cohesive from the magnetic dipolar interactions was calculated relative to the equivalent non-magnetic system of iron oxide and cobalt ferrite (i.e., the two systems become identical after removal of magnetism), by considering mean values of this non-magnetic system (sample size 2000).

Monte Carlo Simulations

The Monte Carlo (MC) simulations were carried out by employing the Metropolis-Hastings algorithm in the canonical ensemble (NVT),[4] according to a coarse-grained interaction model established in a previous study.[3] The interactions included: (i) the van der Waals attraction between NC cores, (ii) magnetic dipole-dipole interaction between NC cores, and (iii) steric repulsion between overlapping oleic acid surfactant chains coating two approaching NCs (note that this potential did not account for any possible steric resistance to shear motion between two NCs). These are all interparticle interactions that will contribute directly to the cohesive energy, i.e., mechanical stability, of the superstructures in question. In addition, the magnetocrystalline anisotropy (MA) potential was included as an intraparticle potential dictating the orientation of the macrospin within each NC, which will have an indirect influence on the cohesive energy through the orientation-dependent magnetic dipole-dipole interactions. Macrospin orientations (steps) were sampled from a normal distributed rotational step size, averaged around zero with standard deviation of 0.01 rad. In this study, the nanoparticle positions were not fixed and thus allowed to fluctuate. Translational steps were also normal distributed: averaged around zero with standard deviation of 0.01 nm. Any possible rotations of the actual NCs were disregarded. The bottom layer of NCs were fixed in the *z*-direction to pin them down on an imaginary surface (which could be any material or liquid subphase), however, no interactions between the NCs and the surface were included in the model. The simulations were performed at RT, unless stated otherwise, with RT parameters.

All simulations started with an initial rotational relaxation of 10^4 steps, in order to mimic the experimental procedure in the initial part of the simulation; nanoparticles were initially embedded in an oleic acid film which was washed off with ethanol after the magnetic field had been switched off (see the Experimental Section above). In the oleic acid film, we expected the nanoparticle positions to remain more static than in air/vacuum, and therefore we considered only rotational steps in this initial part. Following in the main part of the simulation, 100 rotational steps were performed for the macrospins of each NC in the superstructure, then followed by 100 translational steps for each NC, and then repeating this procedure until a total number of $5 \cdot 10^5$ steps was reached both rotationally and translationally. System energies were calculated and dumped after every 100 rotational and translational steps. Mean cohesive energies corresponding to the thermal equilibrium plateau (Figure 2 in the Main Text) were calculated by averaging over the last 2000 data points of the simulation. The superstructure systems simulated at 0 K (Figure S15 and S16 below) were relaxed at 0 K from the initial state

during the course of $5 \cdot 10^4$ rotational steps. The interparticle distance was in the 0 K simulations set to that of the interaction potential minimum (occurring at one complete oleic acid chain length overlap) between two neighboring cubes and left translationally static during the simulations.

The two material systems considered in this study, namely iron oxide (Fe_3O_4) and cobalt ferrite ($CoFe_2O_4$), both exhibit the inverse spinel crystal structure with the same lattice constant (8.39 Å).[5] The bulk saturation magnetization of these two materials are also of similar magnitude: specifically, $4.8 \cdot 10^5$ A m^{-1} for iron oxide,[6] and $4.2 \cdot 10^5$ A m^{-1} for cobalt ferrite.[7] The magnetic moments of the NCs used in the simulations were obtained by multiplying the bulk saturation magnetizations of the respective materials with the nanoparticle volume (i.e., 12^3 nm^3, assuming perfect cubes), followed by a multiplication of a correction factor to account for the disordered surface layer in the 12 nm cubes.[8] We used the same correction factor of 0.9 in this work as in the previous work, which was also supported by experimental magnetic measurements.[2] The MA is notably different for the two materials. In iron oxide, the (bulk) anisotropy constant was reported in the literature to be $-1.3 \cdot 10^4$ J m^{-3},[9-10] whereas for cobalt ferrite, different values were reported (in the range $1.8 \cdot 10^5 – 3.9 \cdot 10^5$ J m^{-3}) depending on which lattice site the cobalt atom occupies in the inverse spinel crystal structure during synthesis.[11-12] In this study we chose an anisotropy constant of $2.6 \cdot 10^5$ J m^{-3} for cobalt ferrite. Cobalt ferrite nanoparticles have been shown in different studies to exhibit a (2-3 times) higher anisotropy constant than the bulk value,[13-14] however, we stress that increasing the anisotropy constant in the simulations will not affect the results much, since the cobalt ferrite nanoparticles already are blocked at RT. Furthermore, the Hamaker constant, dictating the strength of the van der Waals interaction, was found in the literature to be $21.0 \cdot 10^{-20}$ J for magnetite (in vacuum/air).[15] The Hamaker constant of cobalt ferrite is not precisely know,[16] however, owing to the similar properties compared with iron oxide, we assume the Hamaker constant of these two materials to be equal in order to draw comparisons from the simulations solely based on their magnetic behavior. Hence, the iron oxide and cobalt ferrite materials are ideal model systems for studying the effect of magnetism on their superstructure mechanical properties.

Simulations of permalloy systems (Figure S10 and S13 below) were performed by using the same parameters as the iron oxide systems, except for the saturation magnetization, found in the literature to be $8.6 \cdot 10^5$ A m^{-1},[17] and the anisotropy constant which was set to zero.[18] The NC positions were in these simulations fixed, for a qualitative proof of concept as far as dipolar interactions are concerned.

Derivation of Equation (2) and (3)

Derivation of Equation (1) in the Main Text has already been done in a previous work.[3] The derivation of Equation (2) (Main Text) follows below:

$$\begin{cases} E_{\mathrm{coh}} = b\exp\left(a\frac{1}{A}\right) \\ A = \frac{h}{n} \\ a = a_1\frac{1}{n} \\ b = b_1\frac{1}{n} + b_2 \end{cases}$$

$$\Updownarrow$$

$$E_{\mathrm{coh}} = \left(\frac{b_1}{n} + b_2\right)\exp\left(\frac{a_1}{n}\frac{n}{h}\right)$$

$$\Updownarrow$$

$$E_{\mathrm{coh}} = \left(\frac{b_1}{n} + b_2\right)\exp\left(\frac{a_1}{h}\right)$$

$$\lim_{\{n,h\}\to\infty} E_{\mathrm{coh}} = b_2 = E_{\mathrm{bulk}}$$

$$E_{\mathrm{coh}} = \left(\frac{b_1}{n} + E_{\mathrm{bulk}}\right)\exp\left(\frac{a_1}{h}\right)$$

At large aspect ratios (i.e., taken at $1/A = 0$), a Taylor expansion leads to the following simplified expression (Equation (3) in the Main text):

$$E_{\mathrm{coh}} = b\exp\left(a\frac{1}{A}\right)$$

$$E_{\mathrm{coh}} \overset{\mathrm{Taylor}}{\approx} b + ab\frac{1}{A} = \frac{b_1}{n} + b_2 + a_1\left(\frac{b_1}{n} + b_2\right)\frac{1}{h}$$

$$E_{\mathrm{coh}} \approx \frac{b_1}{n} + b_2 + \frac{a_1 b_1}{nh} + \frac{a_1 b_2}{h} \approx \frac{a_1 b_2}{h} + \frac{b_1}{n} + b_2$$

$$E_{\mathrm{coh}} \approx \frac{a_1 E_{\mathrm{bulk}}}{h} + \frac{b_1}{n} + E_{\mathrm{bulk}}$$

Supporting Figures, Tables and Discussions

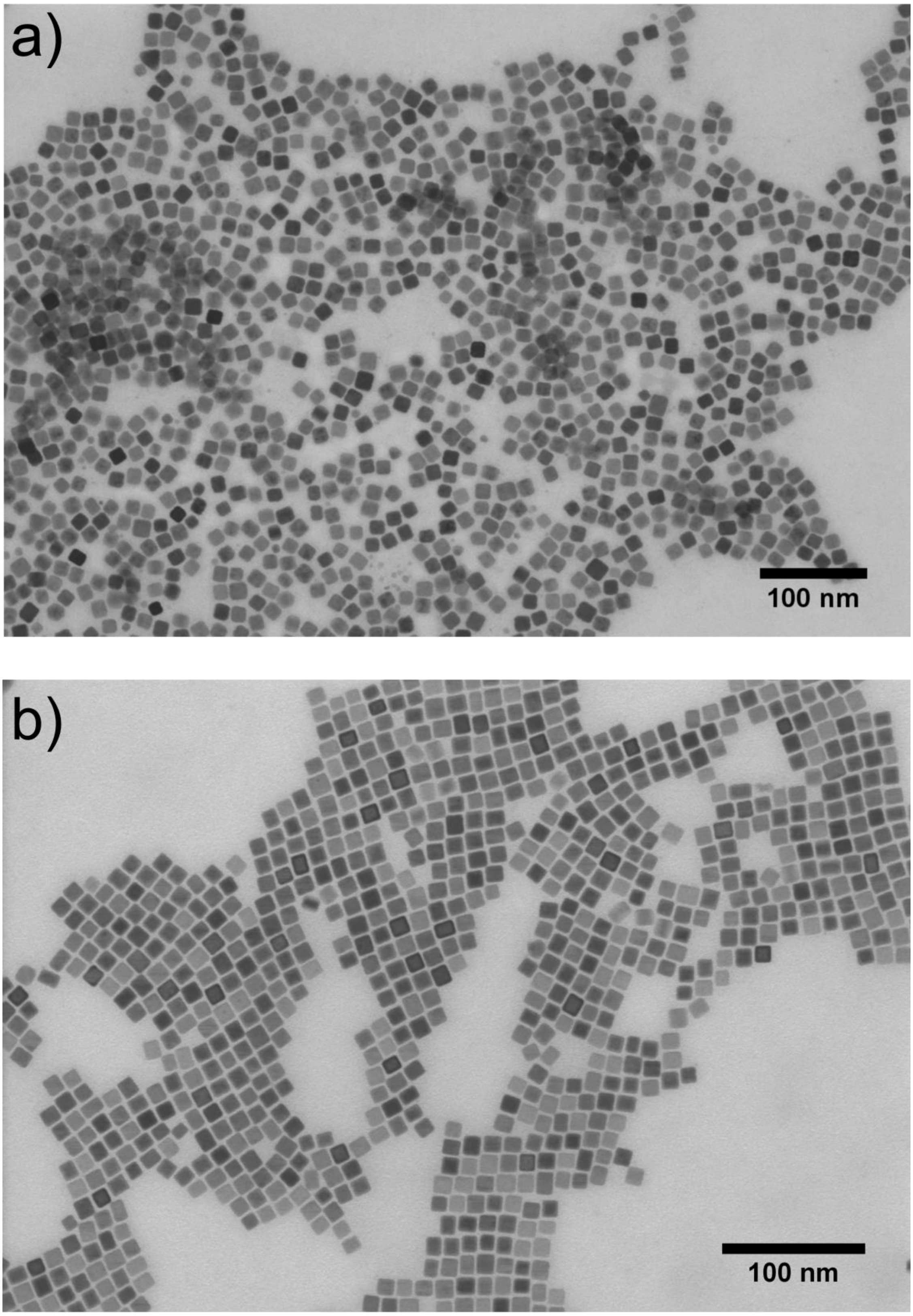

Figure S1. Scanning transmission electron micrographs of the NC distributions. a) Iron oxide NCs. b) Cobalt ferrite NCs.

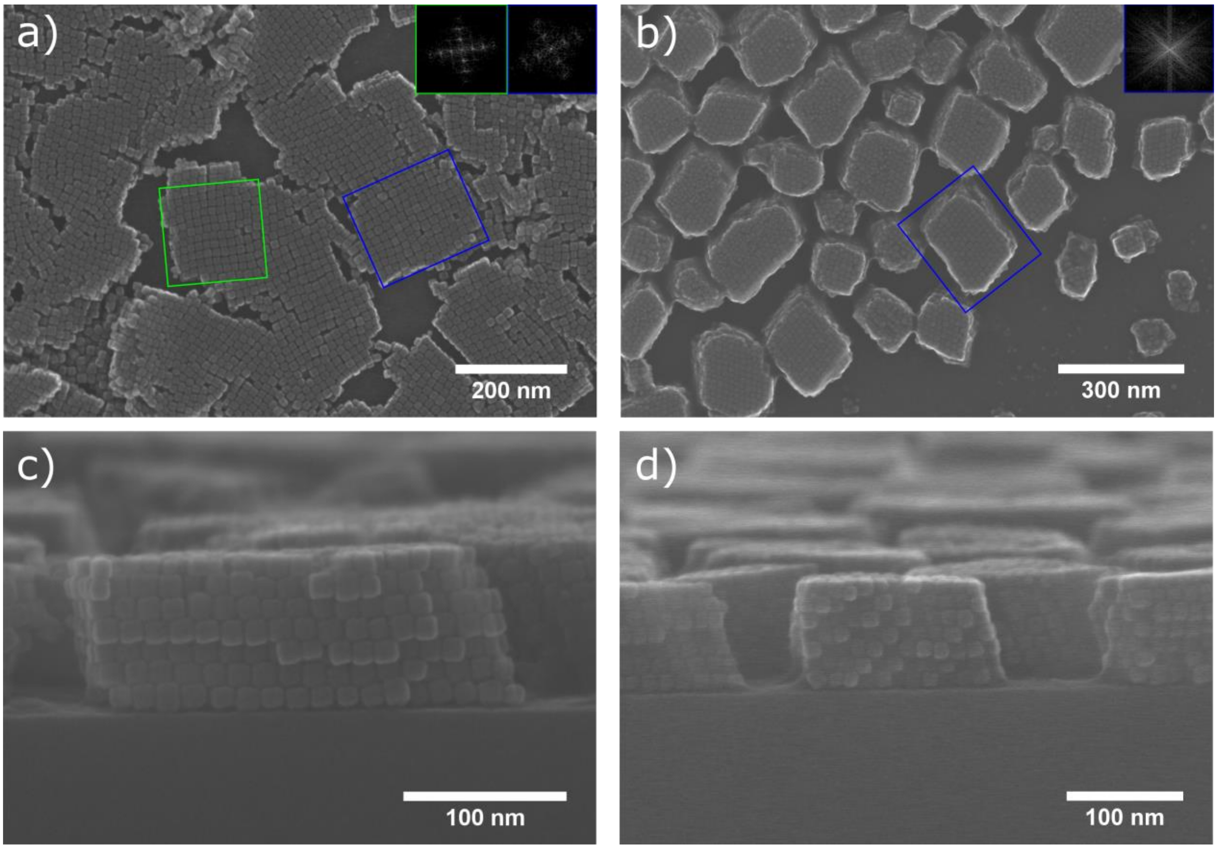

Figure S2. SEM micrographs of simple cubic iron oxide superstructures. a) Top view of few-layer superstructures. b) Top view of pillars. Parts (c) and (d) show cross-sectional images of superstructures. Fast Fourier transforms (FFTs) representing the indicated areas have been added as insets in (a) and (b). The FFTs reflect the presence of simple cubic supercrystals on the samples.

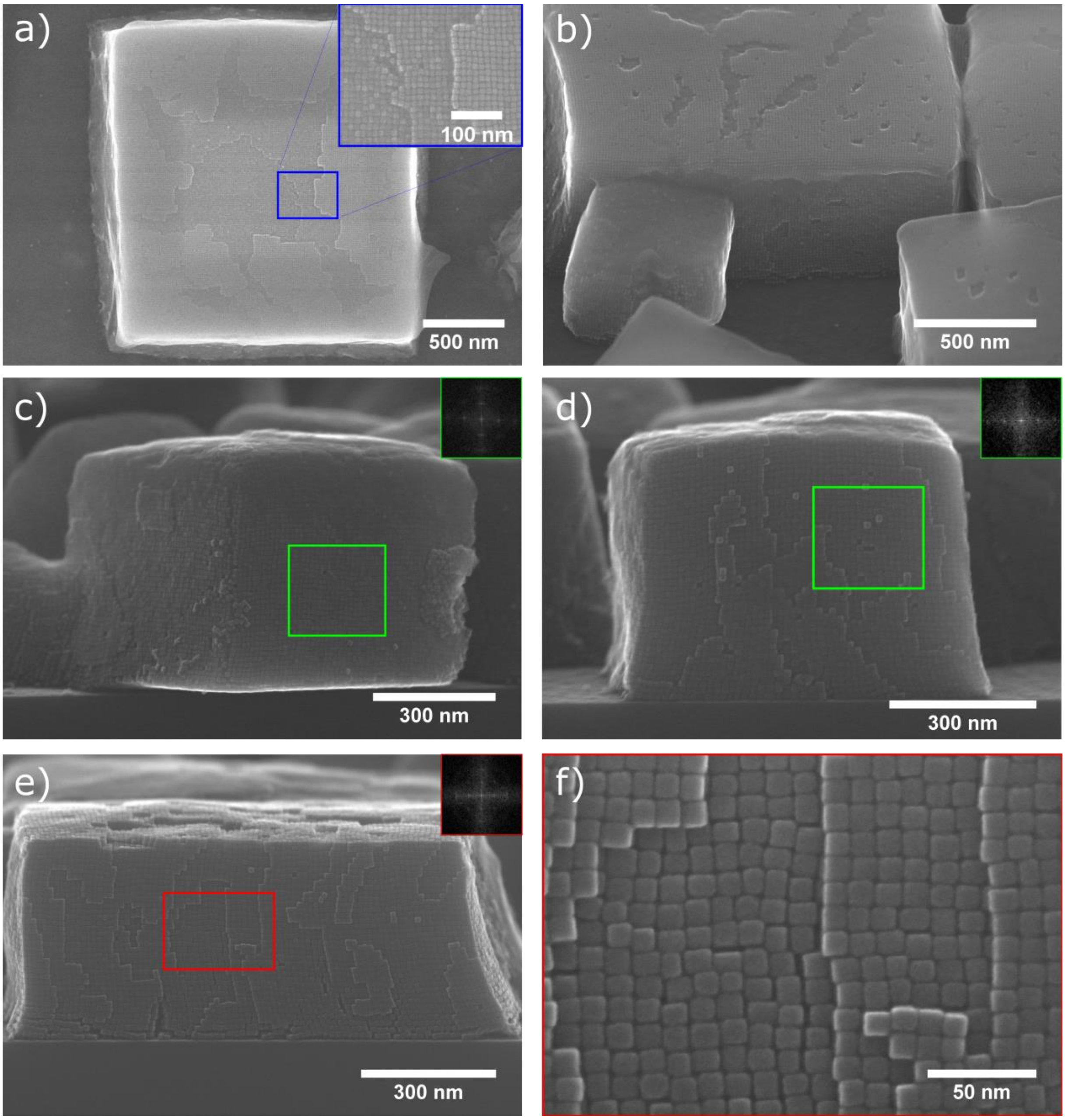

Figure S3. SEM micrographs of simple cubic cobalt ferrite superstructures. a) Top view of a supercube with inset (enlarged blue frame) showing coherent stacking of layers. b) Tilted view (40°). c) A perfectly stacked supercube with straight walls of NCs "hanging over" the scribed silicon substrate cross section. d) A supercube cut in half revealing the simple cubic superlattice of the interior. e) Cross section of a larger superstructure with a high-resolution image in (f) (red frame) showing the coherent stacking of layers. Some distortions and skewness, presumably arising during lift-off, can be seen in (e), especially in the lower right part of the superstructure. FFTs reflecting simple cubic superlattices are shown in the insets of (c)–(e).

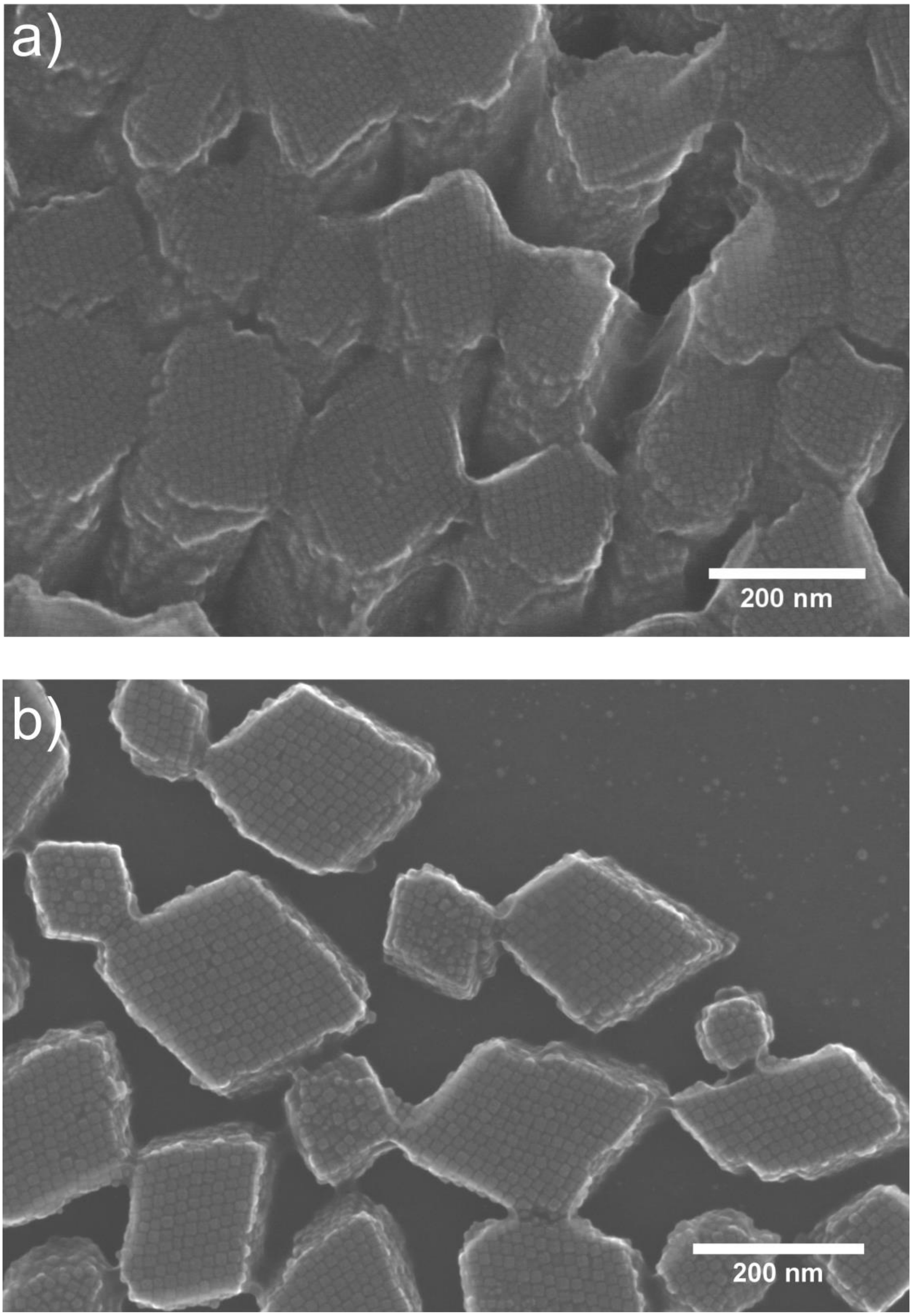

Figure S4. SEM micrographs of iron oxide superstructure polymorphs. Apart from the simple cubic superlattice, iron oxide superstructures exhibiting the a) ⟨111⟩-configuration and b) quasi-hexagonal arrangement, are also observed.

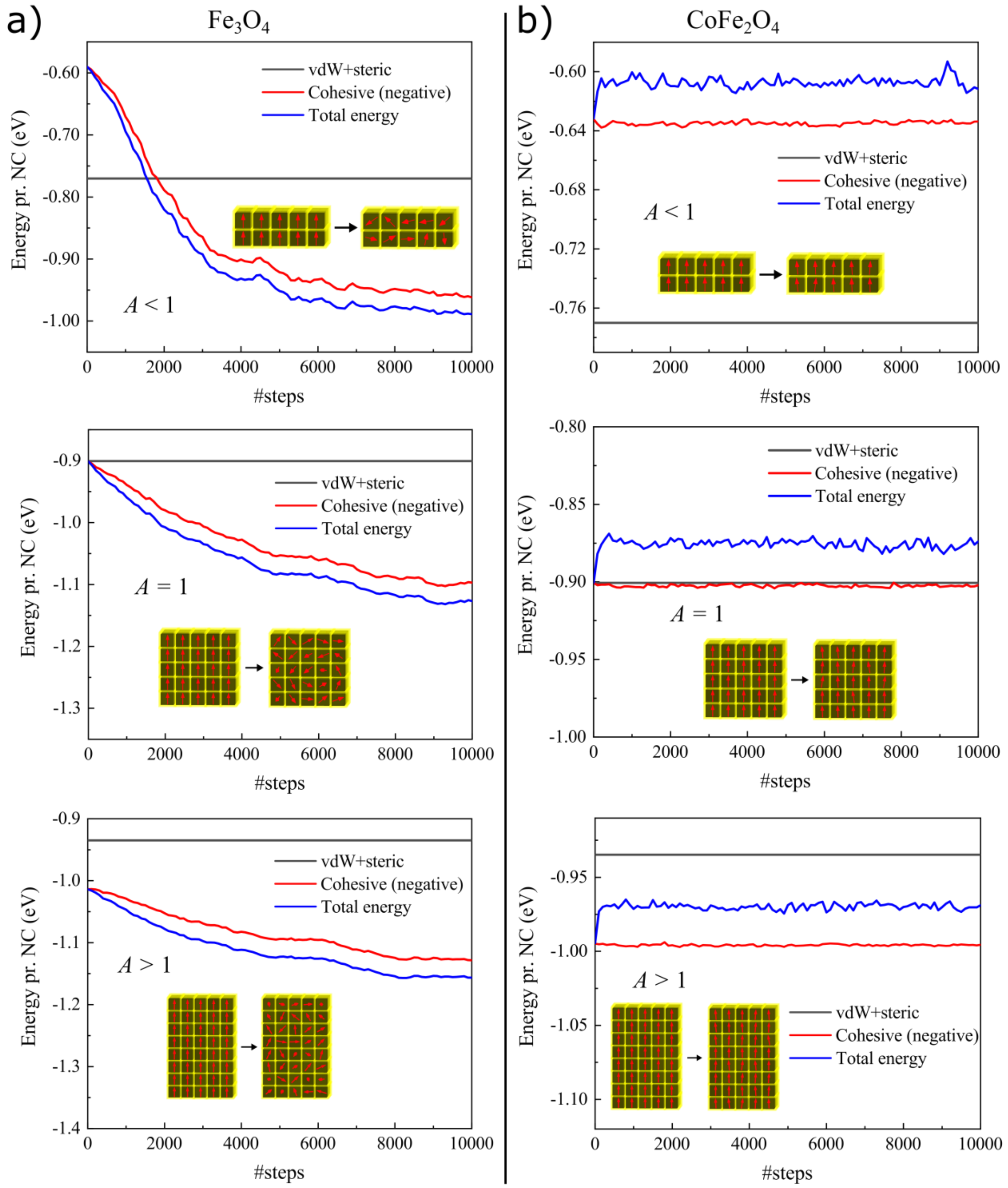

Figure S5. Energy plots of the initial rotational relaxation of 10^4 MC steps for the superstructures corresponding to Figure 2 in the Main Text. a) The macrospins relax in all the iron oxide superstructures, in which the (negative) cohesive energy reaches well below the vdW+steric curve (horizontal line). b) No macrospin relaxation occurs in the cobalt ferrite systems due to the strong MA. Following the translational relaxations after the 10^4 initial steps (not shown here), a positive jump in the energy curve occurs for all superstructures due to instantaneous thermal fluctuations in NC positions, which were originally situated at interparticle potential minimum.

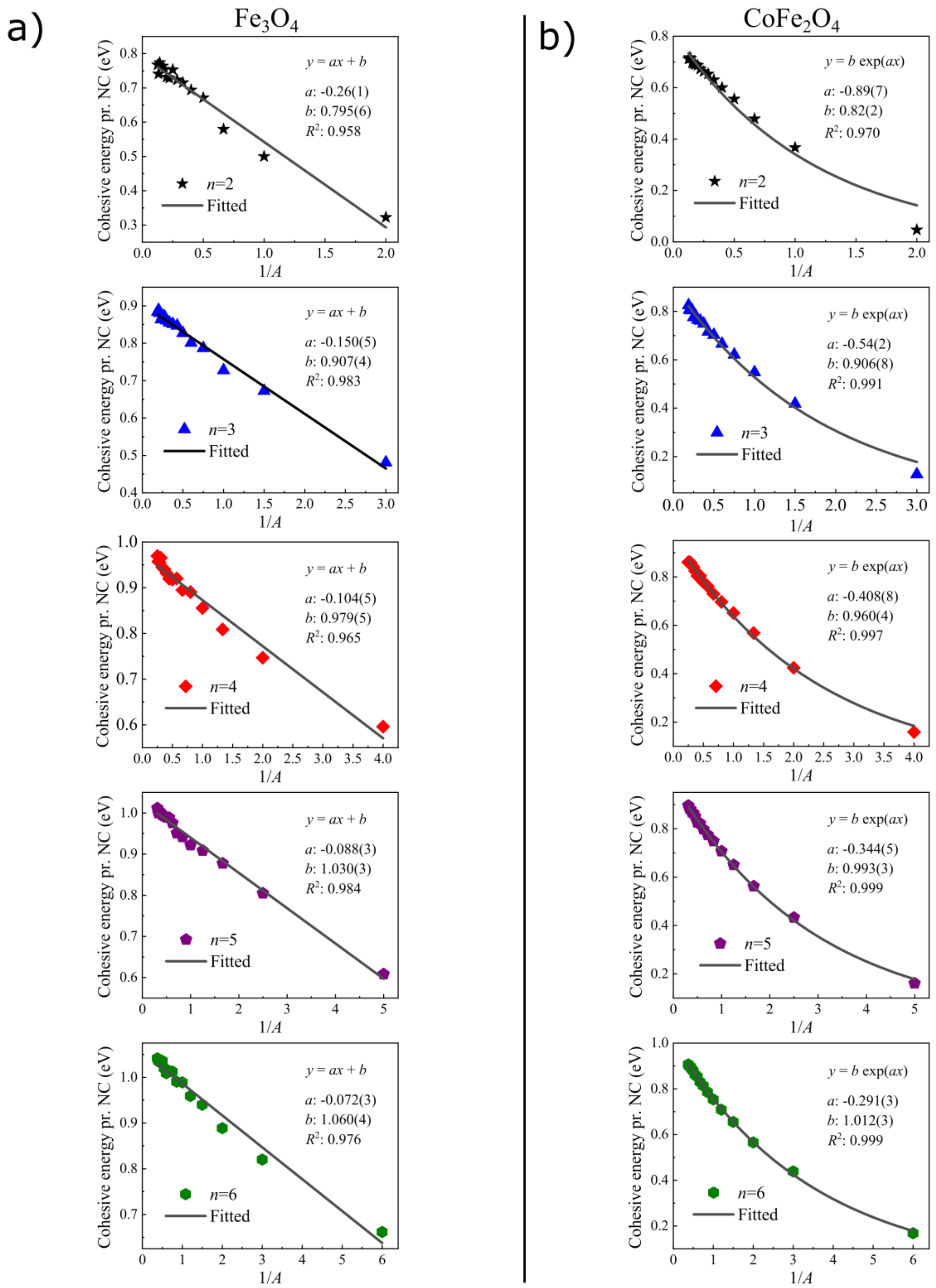

Figure S6. Curve fitting of the cohesive energy data in Figure 3a,b in the Main Text. The datasets corresponding to each *n*-number is shown for the a) iron oxide and b) cobalt ferrite systems, fitted to a linear and exponential function, respectively. The obtained *a* and *b* parameters, with fitting errors in parenthesis, are displayed together with the associated R^2 value. Details regarding linear and exponential curve fitting can be found in the Experimental Section above.

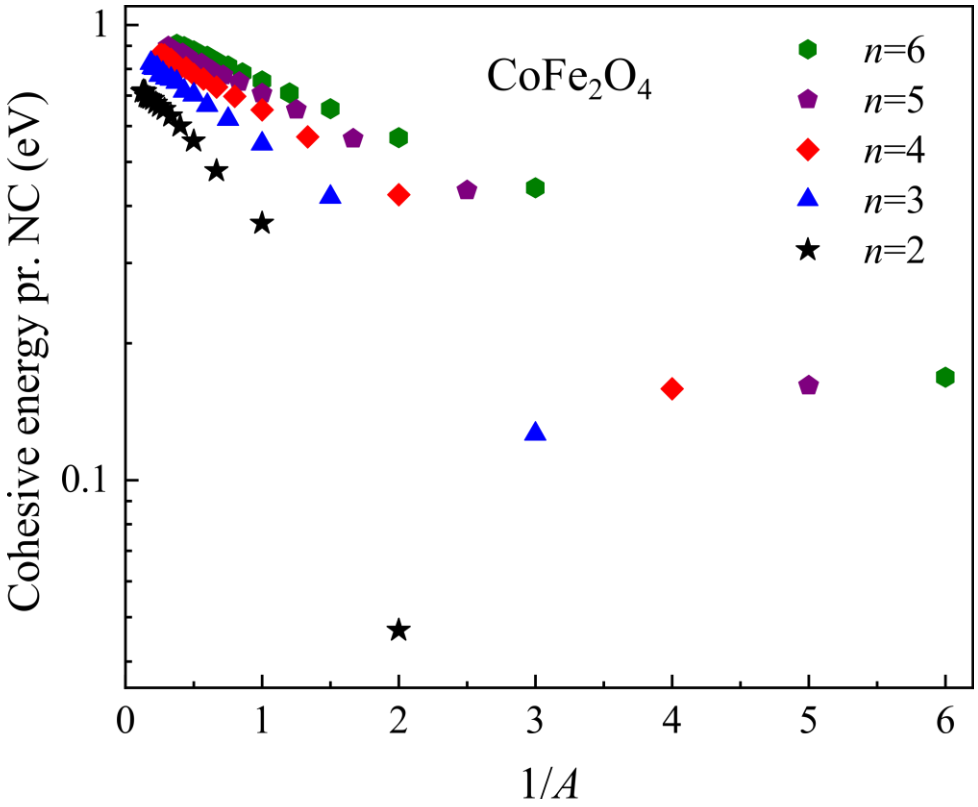

Figure S7. Semi-log plot of the cobalt ferrite mean cohesive energy data of Figure 3b in the Main Text. The plot shows linear relationships with respect to reciprocal aspect ratio (1/*A*), as expected from the proposed exponential dependence. A slight deviation is seen for the superstructures with $n = 2$ (where the original data points also fit well to a linear function), however, the linear trend in the semi-log plot becomes evident as the cross section increases.

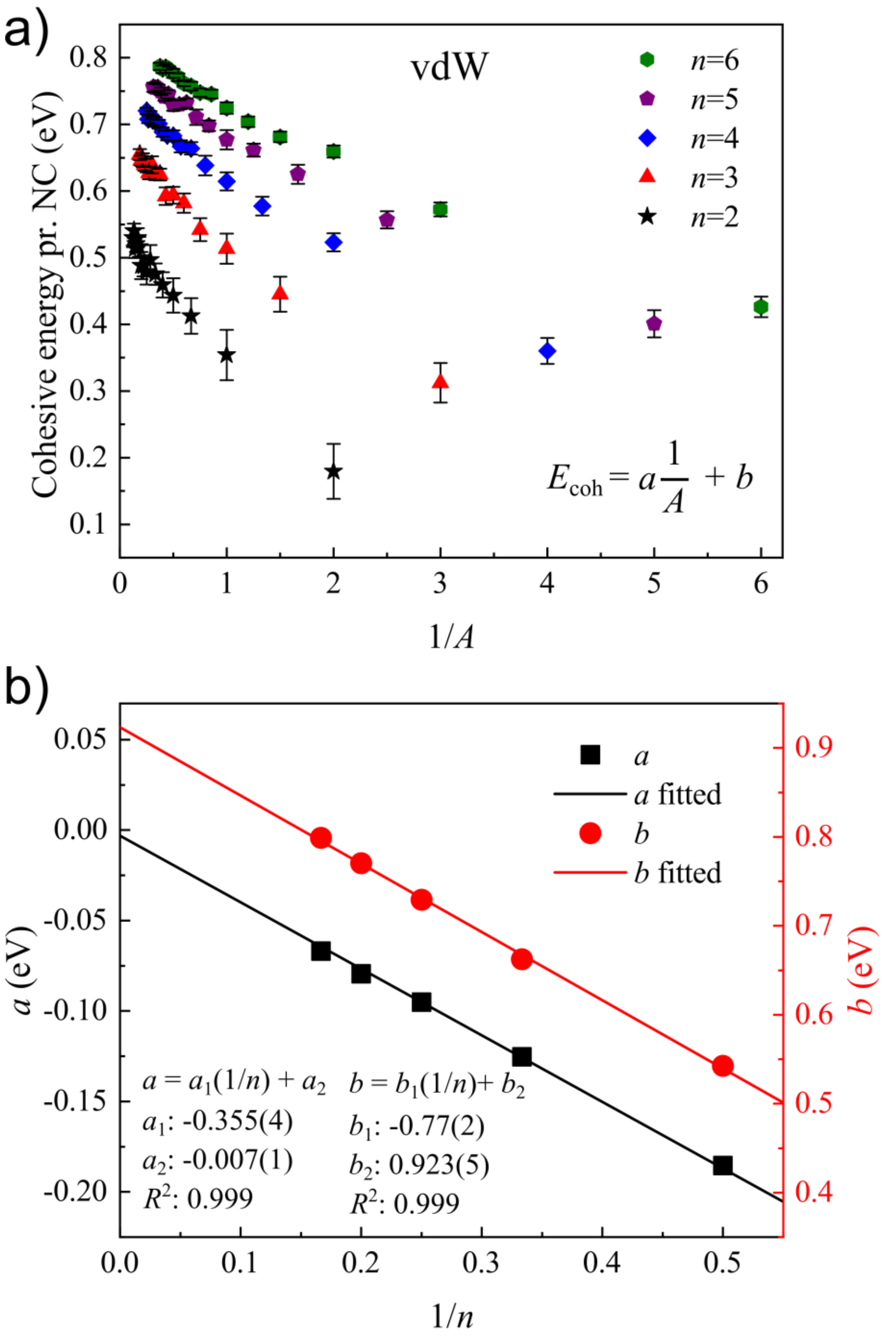

Figure S8. Cohesive energy per NC and curve fittings, obtained from the simulations of the non-magnetic systems. a) Cohesive energy per NC with standard deviations as a function of 1/*A*. A linear relationship is observed in the data points corresponding to superstructures of the same cross section (*n*-number). b) The *a* and *b* parameters obtained for data points corresponding to the same *n*-number, and plotted as a function of 1/*n*, also reveal a linear relationship (obtained fitting parameters in units of eV are displayed together with the corresponding fitting errors in parentheses and R^2 values). The cohesive energy data in (a) are presented as mean ± standard deviation (sample size 2000). Details on linear regression are given in the Experimental Section above.

Near-Zero MA Superstructure Systems

Up to now, our results clearly demonstrate the profound effect of MA on the mechanical stability in the self-assembled superstructures. A weak (⟨111⟩) MA leads to a relatively disordered macrospin configuration at RT, which, in turn, enhances the mechanical stability isotropically, whereas a strong (⟨100⟩) MA may lead to mechanical anisotropy as a consequence of enforced parallel macrospin alignment. However, to further illustrate the role of MA in the superstructure mechanical properties, we remove the MA from the iron oxide systems (i.e., MA = 0; no intraparticle driving force influencing macrospin alignment). Interestingly, shape anisotropy becomes evident for macrospin alignment in such zero-MA iron oxide structures of high *A*, as shown in Figure S9 below. Specifically, in the absence of MA, the effect of shape in high *A* superstructures tends to align the macrospins in a super-antiferromagnetic pattern to maximize vertical head-to-tail and side-by-side antiparallel dipole-dipole interactions (reducing the demagnetizing field). Hence, shape anisotropy could impose macrospin ordering and lead to an isotropic mechanical stabilization in magnetic superstructures with a low enough MA-to-dipolar energy ratio. An experimental candidate for such scenario is permalloy ($Ni_{80}Fe_{20}$), with a very low MA and a saturation magnetization about twice as large as that of iron oxide (yielding magnetostatic interactions four times stronger).[17-18] In an equivalent permalloy superstructure system, thermal energy fluctuations are suppressed to a greater extent by the strong dipolar interactions, thus providing a higher degree of alignment at RT (Figure S10). The cohesive energy per NC is in general found to be slightly larger at RT for iron oxide with zero MA (cohesive energy plot in Figure S11), compared with the real system with non-zero MA (MA $\neq$ 0), as expected from the higher "freedom" of the system in the zero MA case to minimize the demagnetizing field through spin relaxation. However, for the shape anisotropy to have an effect, the aspect ratio of a superstructure should be high enough (depending on the thermal energy and the strength of the dipole-dipole interaction, e.g., $A > 2$ for iron oxide at RT). For $A \approx 1$, no definite ordering (other than order of a short ranged and short termed nature) is observed during the entire course of the simulation for the zero MA iron oxide systems (Figure S12), as well as in the permalloy simulations (Figure S13). The same holds true for structures of $A < 1$, however in-plane macrospin alignment is favored for monolayers (Figure S13 and S14), which is also an obvious consequence of shape anisotropy.

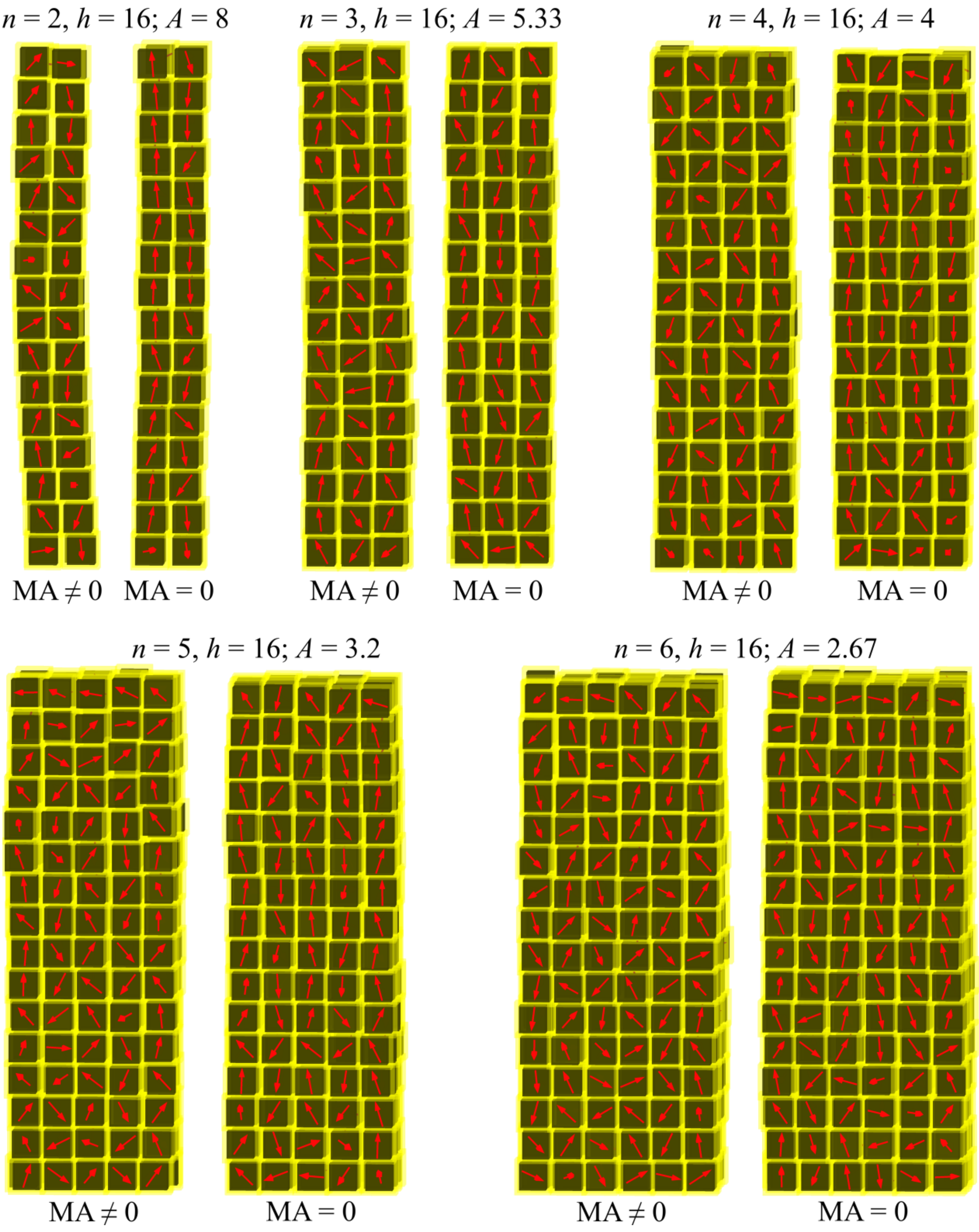

Figure S9. Snapshots of high *A* superstructures of iron oxide in thermal equilibrium, both with and without MA. A significant shape-induced vertical alignment is shown for the zero MA superstructures. As the superstructure aspect ratio reaches below 3 (i.e., for n = 5, h = 16), the effect of shape anisotropy is seen to become less profound.

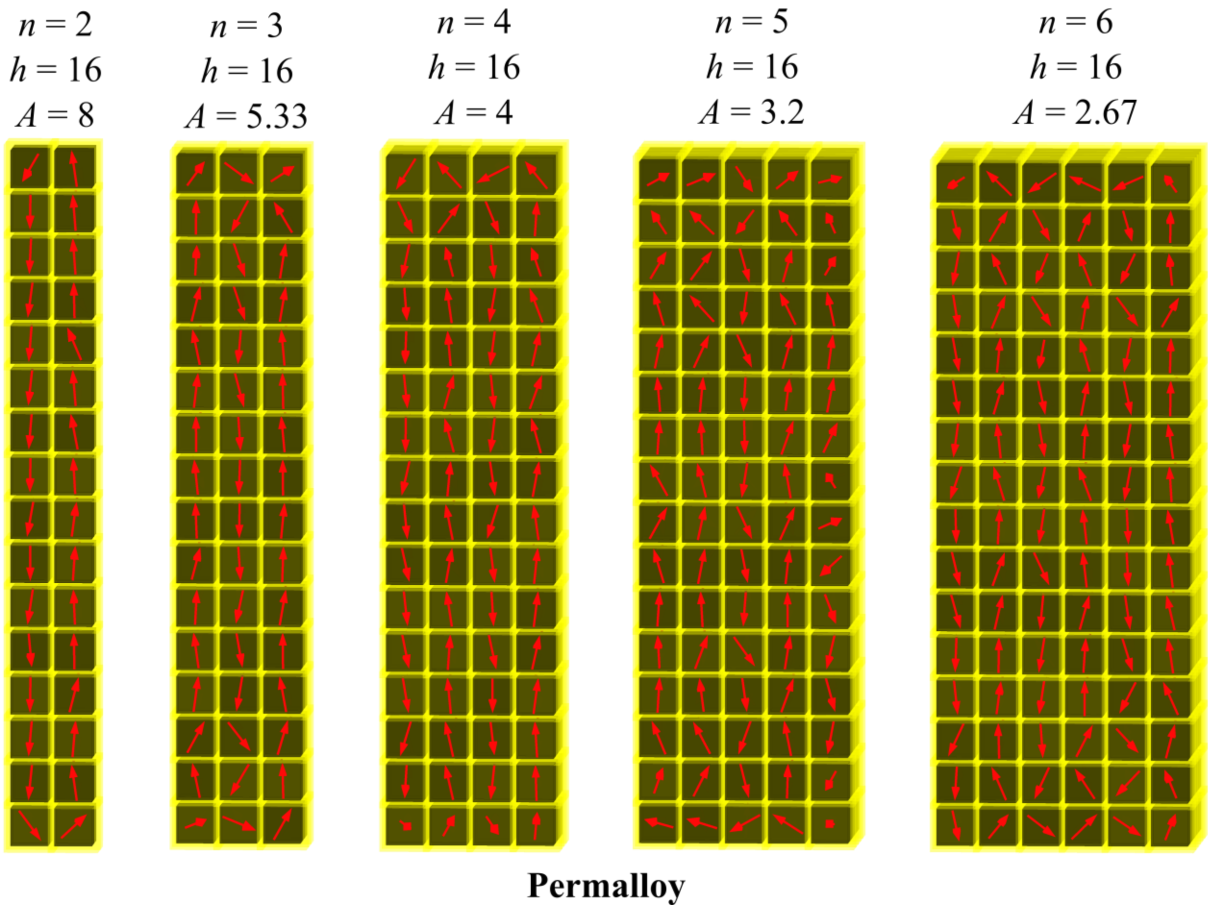

Figure S10. Snapshots of high *A* superstructures of permalloy in thermal equilibrium. Since the magnetization of permalloy is about twice the value of iron oxide, the magnetic dipolar interactions (four times stronger than for iron oxide) in the absence of MA are strong enough to maintain a high degree of ordering at RT (i.e., in high *A* structures where shape anisotropy is significant). Hence, permalloy is recognized as an experimental candidate to realize stable 3D super-antiferromagnetic materials at RT.

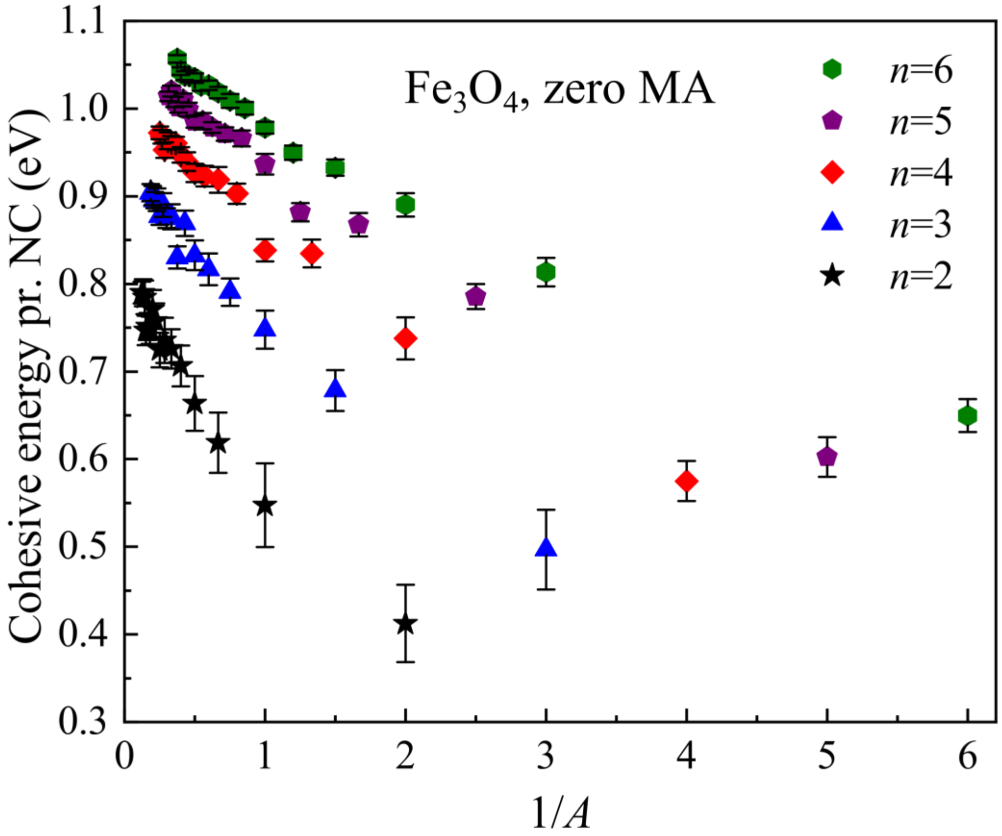

Figure S11. Plotted cohesive energy per NC as a function of 1/*A* for the iron oxide systems of zero MA (at RT). Also in the zero MA case, linear trends are observed. The cohesive energy data are presented as mean ± standard deviation (sample size 2000).

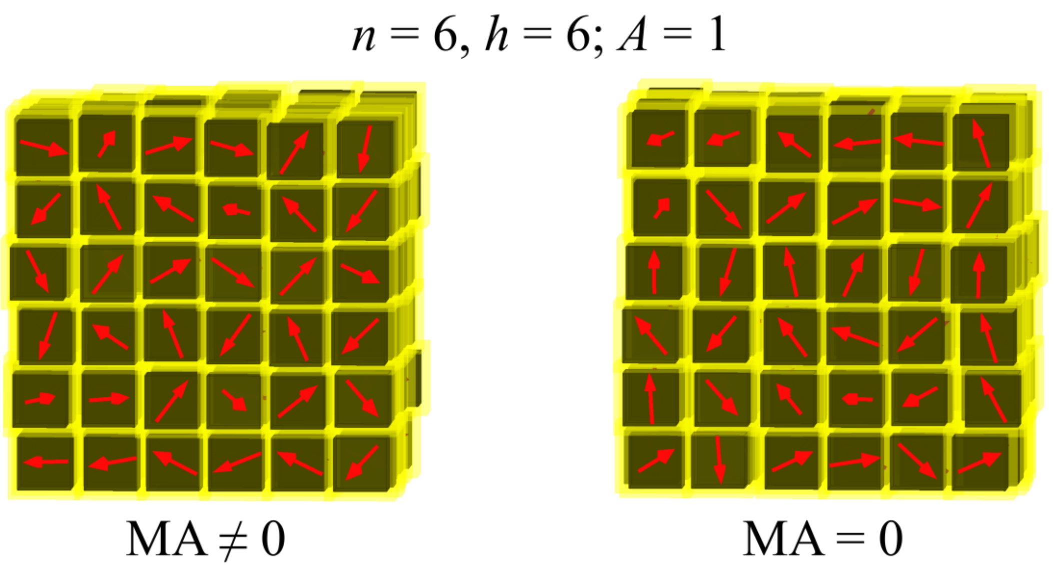

Figure S12. Snapshots of iron oxide superstructures with A = 1 (n = h = 6) in RT thermal equilibrium, both with and without MA. No effect of shape anisotropy is observed in this case, indicating no substantial difference between real iron oxide and zero MA iron oxide.

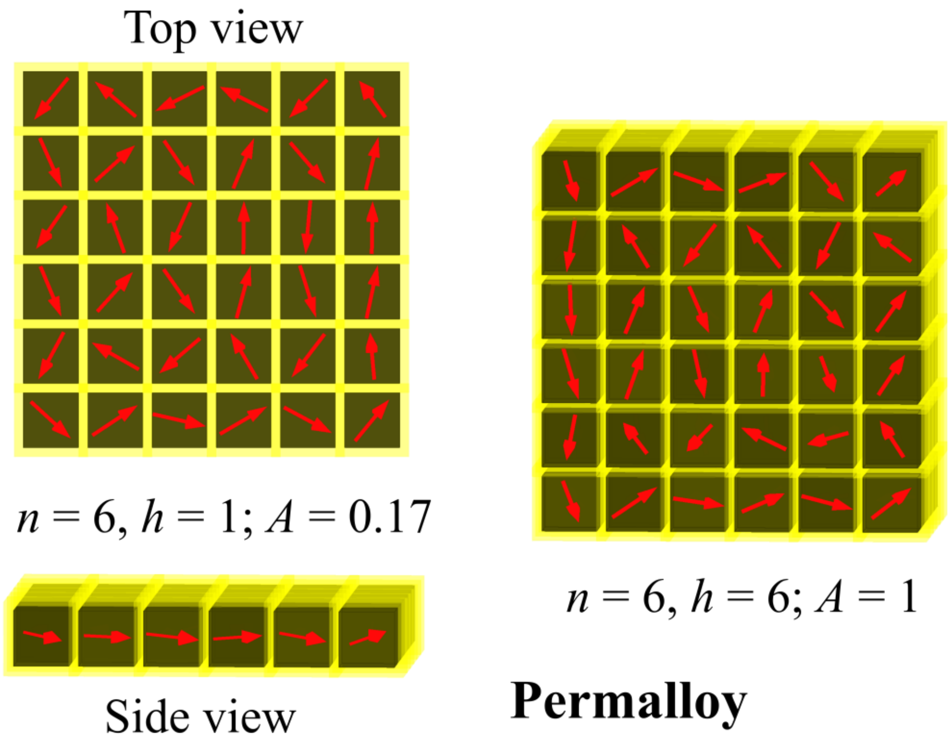

Figure S13. Snapshots of two superstructures of permalloy in RT thermal equilibrium: one monolayer and one supercube (A = 1).

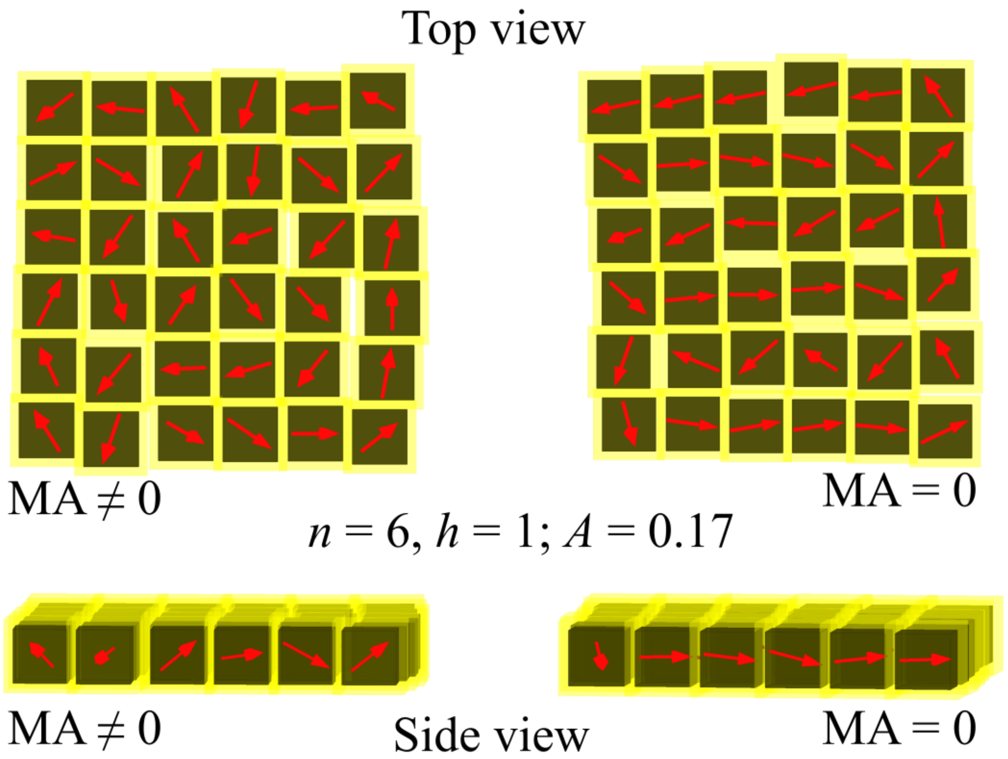

Figure S14. Snapshots of low *A* superstructures of iron oxide in RT thermal equilibrium, both with and without MA. The snapshots, both in top and side view, show how shape anisotropy (in the absence of MA) tend to align the spins in-plane to cancel out the demagnetizing field. In the zero MA case, a super-antiferromagnetic ordering can be observed in this snapshot, however, the direction of the alignment fluctuates between the *x*- and *y*-axis as time progresses.

Relative Mechanical Stability of Superstructures of Different Macrospin Configurations

The actual macrospin configuration (via magnetic anisotropy) of the system can clearly act as another means of tailoring mechanical properties, a possibility which is non-existent in non-magnetic systems. It is therefore important to determine the relative mechanical stability of such magnetic superstructure systems of different spin configuration. By eliminating the effect of thermal energy upon cooling to 0 K, we identify three differently ordered spin configurations resulting from the systems considered above (note that these are not necessarily the ground state configurations): SFM, super-antiferromagnetic and a spin-ice-like (i.e., *super*-spin-ice) phase. In our recent work, we also showed that the iron oxide superstructures freeze into spin-ice-like patterns upon cooling to 0 K.[3] The spin-ice-like configuration results from a competition between the magnetic dipolar interactions and the MA aligning the spins close to the diagonal easy axes (effectively canceling out the demagnetizing field). In the case of the cobalt ferrite systems, the structure becomes a perfect SFM at 0 K, where the corresponding saturated demagnetizing field is sustained by the strong anisotropy along the $\langle 100 \rangle$ easy axes. Furthermore, if the MA is effectively removed at 0 K, the spins align into a super-antiferromagnetic pattern with flux-closure patterns at the ends of the superstructure (Figure S15). We compare these three different 0 K scenarios (Figure S16) in which the spins have been relaxed at 0 K with the NC interparticle distance assumed to be one oleic acid chain length, at the interparticle potential minimum (see section above on Monte Carlo Simulations for more details). The demagnetizing field has been effectively canceled out in the spin-ice-like structure as well as in the super-antiferromagnetic structure, leading to an isotropic mechanical stabilization in both cases. In the SFM case, the structure is a perfect permanent magnet and should show a mechanical anisotropy similar to the RT case (i.e., destabilized in-plane and stabilization along the vertical direction). Overall, in terms of the cohesive energy per NC, the mechanical stability of superstructures displaying these different types of spin ordering is summarized as follows: SFM < spin-ice-like < super-antiferromagnetic (provided the magnetization, and thus the dipolar interactions, are of similar magnitude in all cases). At RT, a snapshot of the disordered superparamagnetic configuration at any given time will fall between the SFM and super-antiferromagnetic configurations (and, as previously established in the literature, will be marginally less mechanically stable than the spin-ice[3]).

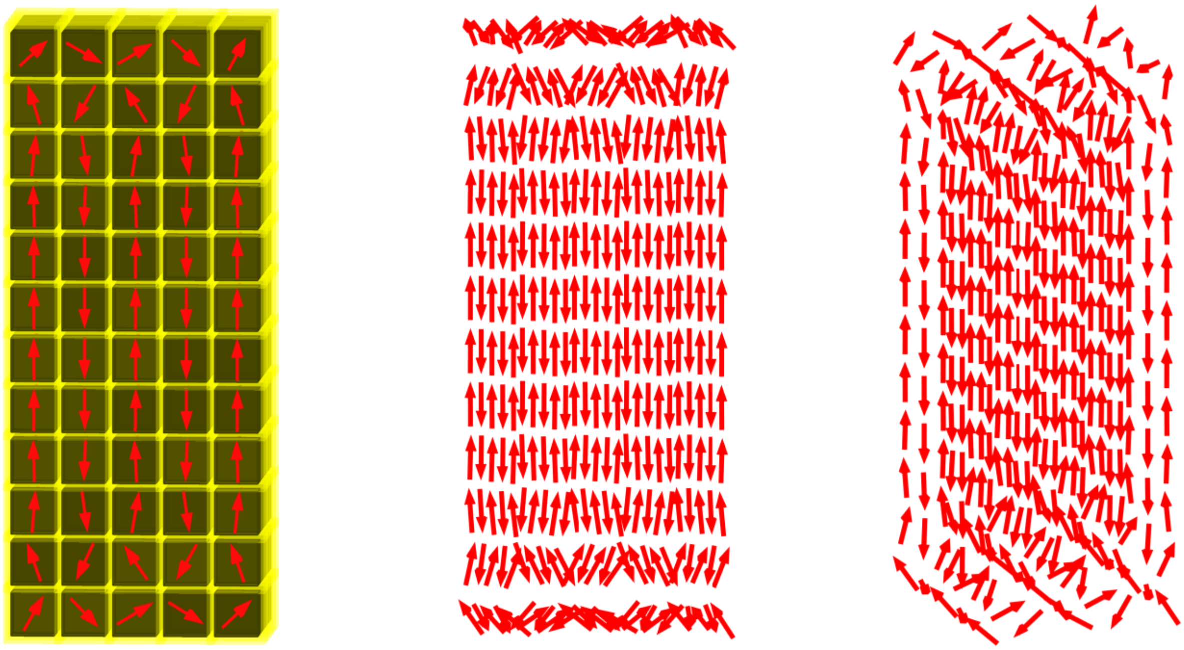

Figure S15. Spin relaxation at 0 K in a high *A* iron oxide superstructure with zero MA, yielding a super-antiferromagnetic macrospin alignment with flux-closure patterns at the ends. The 0 K macrospin state is shown at different angles, both with and without the actual NC body included.

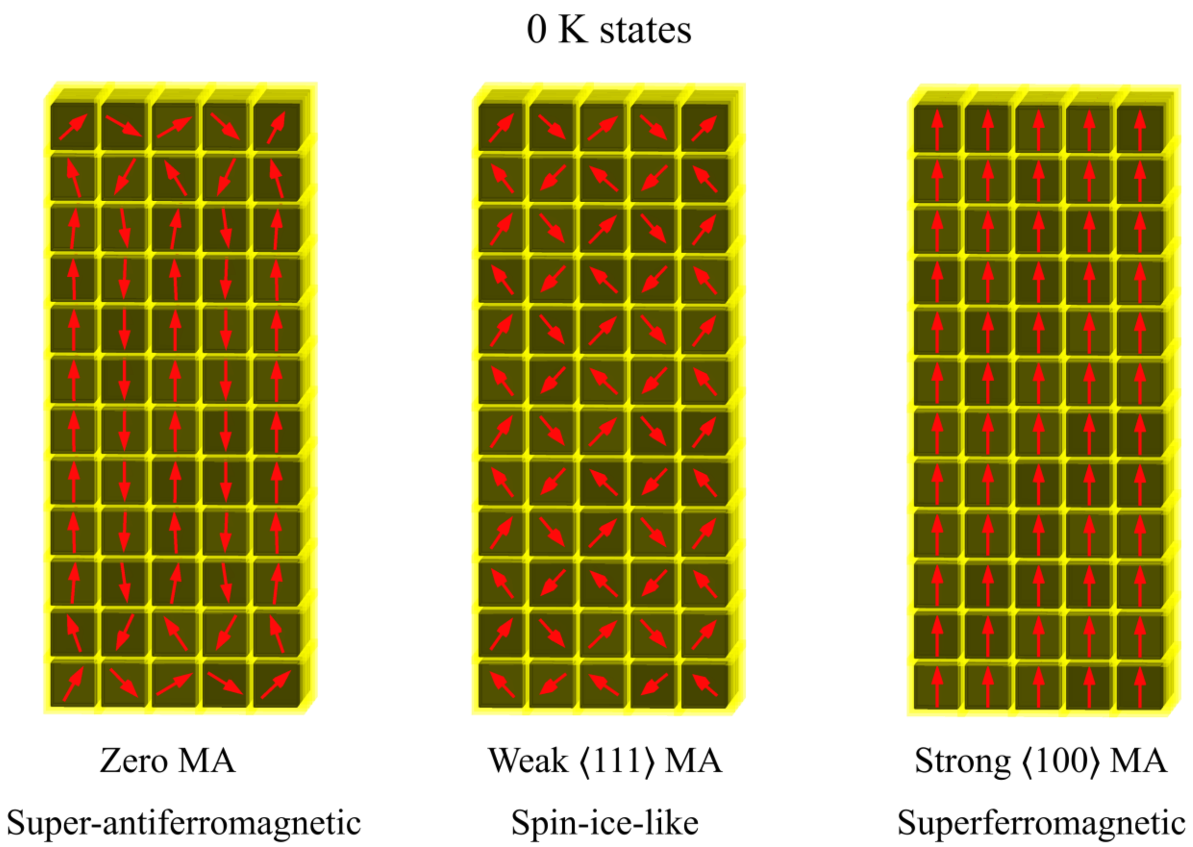

Figure S16. Simulated cooling to 0 K in superstructures with MA of different strength and symmetry. When the MA is near zero, the macrospins organize themselves into a super-antiferromagnetic alignment in which the ends are closed off by flux-closure patterns to effectively suppress the demagnetizing field. For a soft magnetic system of cubic symmetry and with easy axes along the ⟨111⟩-directions (e.g., iron oxide), the 0 K state is spin-ice-like. Strong MA of cubic symmetry along the ⟨100⟩-directions (e.g., cobalt ferrite) yields a metastable superferromagnetic alignment. The overall mechanical stability of superstructures displaying these types of macrospin configurations is summarized as follows: superferromagnetic < spin-ice-like < super-antiferromagnetic.

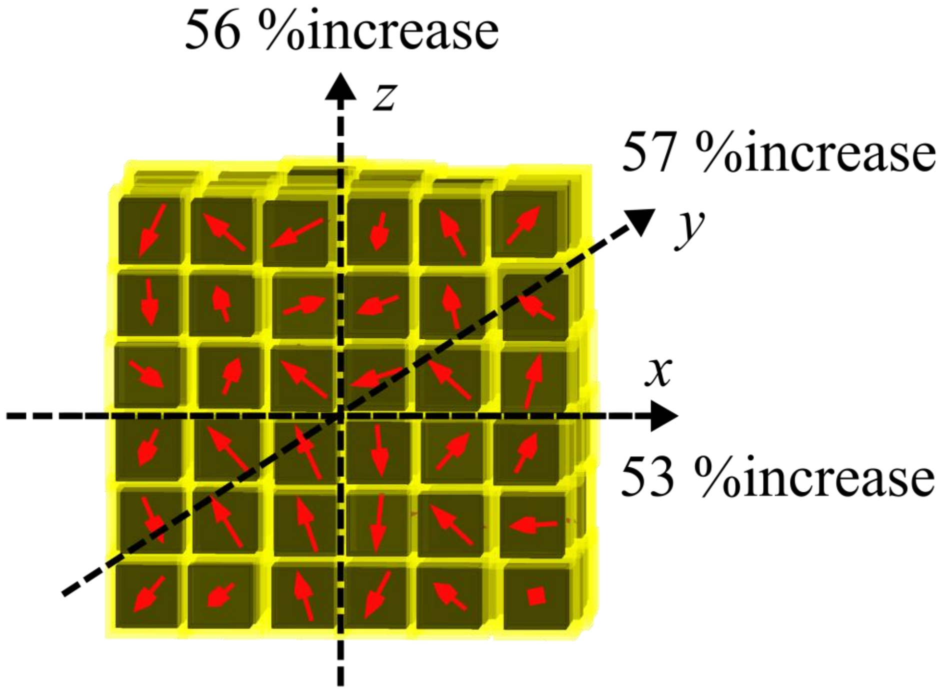

Figure S17. The %increase in cohesive energy from the magnetic dipolar interactions calculated along the different Cartesian axes of an iron oxide supercube (n = h = 6; A = 1). As expected from a mechanically "isotropic" system, the superstructure displays values of similar magnitude along the three different axes: 53%, 57% and 56% (this small difference is expected to disappear for larger systems, or for longer simulations with averaging over a higher number of data points). Note that these values are slightly larger than the overall %increase of the same superstructure, reported in Figure 3c in the Main Text to be ≈37%. If we also take the other (low packing density) axes into account, like, e.g., the diagonals, the %increase will diminish towards this value.

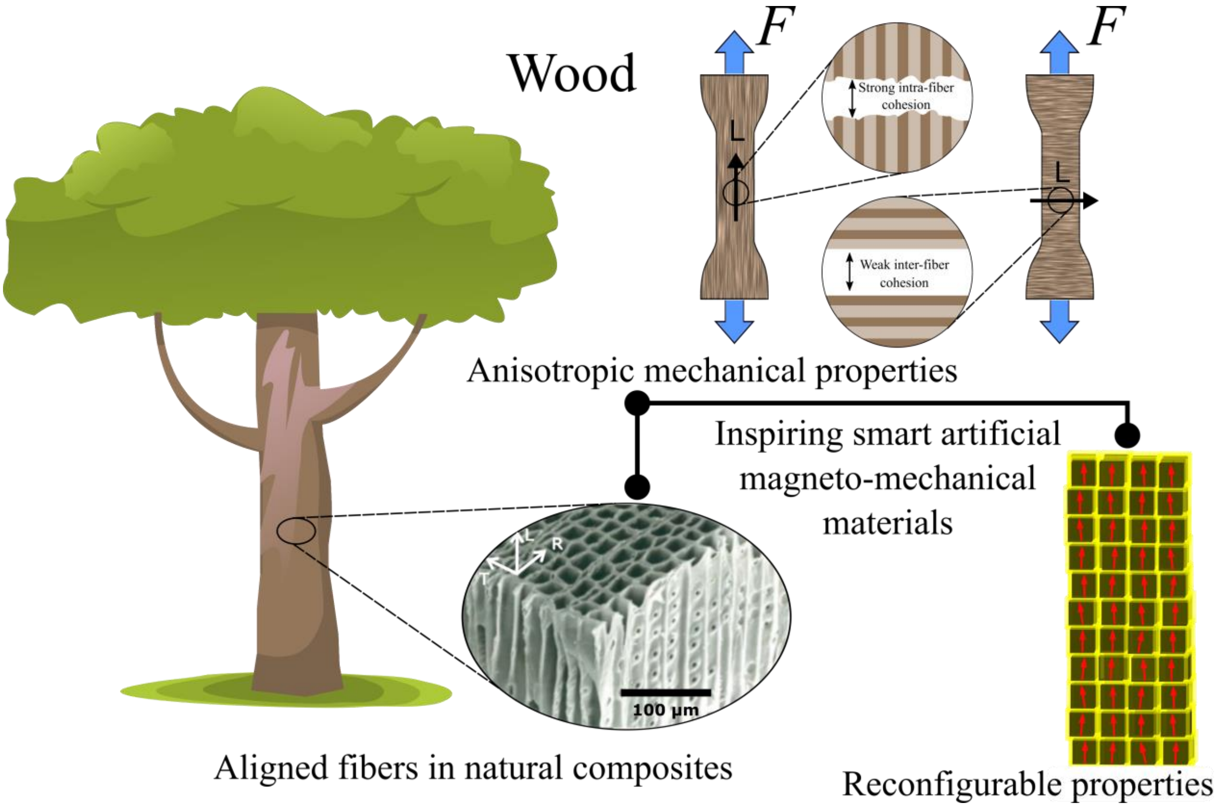

Figure S18. Comparison of the anisotropic mechanical properties of wood and artificial magnetic superstructures. The aligned macrospins of mechanically anisotropic magnetic superstructures mimic the fibrous microstructure of natural composites, commonly found in plants and trees. One example is the familiar wooden microstructure where the strong uniaxial fibers serve to maintain the mechanical integrity of the tree, making wood a very attractive material for structural engineering.[19] Wooden microstructures can therefore inspire the design and creation of smarter artificial materials where such a high-strength axis (analogous to the wooden microfibers) could be remotely reconfigured as desired (Figure 4, Main Text), to effectively sustain loads from different directions during operation. The SEM image in the figure shows magnified wooden microfibers, in which the coordinate axes, L, R and T, denote the longitudinal, radial and tangential axes with respect to the wooden microstructure (i.e., fiber direction). The SEM image is adapted with permission.[19] Copyright 2015, Elsevier.

Table S1. Average interparticle spacings along the Cartesian axes for the largest systems considered in this study (i.e., $n = 6$, $h = 16$) of iron oxide, cobalt ferrite and equivalent non-magnetic NCs.[a)]

Material System	*x*-axis [nm]	*y*-axis [nm]	*z*-axis [nm]
**Fe_3O_4**	2.89(4)	2.69(4)	2.28(2)
**$CoFe_2O_4$**	3.22(5)	3.38(5)	2.20(1)
**Non-magnetic**	3.09(5)	2.98(4)	2.40(3)

[a)]The mean value is given, with the standard error in parentheses referring to the least significant digit of the corresponding value. The average interparticle spacing in the superferromagnetic cobalt ferrite superstructure is observed, as expected, to be smaller along the *z*-axis and larger along the *x*- and *y*-axis than that of the iron oxide system, reflecting the stronger attractive vertical and repulsive in-plane dipolar interactions (compared with superparamagnetic iron oxide with weaker isotropic attractive interactions). Furthermore, the in-plane spacings in the non-magnetic system fall in-between the values of the magnetic systems, whereas the spacing along the *z*-axis is observed to be larger due to the absence of magnetic attractions. Note that there is a smaller mean interparticle spacing in the isotropic systems along the *z*-axis relative to the *x*- and *y*-axis, which is attributed to the larger size (i.e., $h > n$), and thus the higher stability, along this axis, as well as the *z*-axis-pinning of the bottom NC layer (see the Monte Carlo Simulations section above) which suppresses the freedom of movement along this axis to some extent.

References

[1] G. Singh, H. Chan, A. Baskin, E. Gelman, N. Repnin, P. Král, R. Klajn, *Science* **2014**, *345*, 1149.

[2] V. Håkonsen, G. Singh, J. He, Z. Zhang, *Mater. Horiz.* **2018**, *5*, 1211.

[3] V. Håkonsen, G. Singh, P. S. Normile, J. A. De Toro, E. Wahlström, J. He, Z. Zhang, *Adv. Funct. Mater.* **2019**, *29*, 1904825.

[4] D. P. Kroese, T. Taimre, Z. I. Botev, *Handbook of Monte Carlo Methods*, John Wiley & Sons, Inc., Hoboken, NJ **2011**.

[5] K. Ali, Y. Javed, Y. Jamil, in *Complex Magnetic Nanostructures: Synthesis, Assembly and Applications* (Ed: S. K. Sharma), Springer International Publishing, Cham **2017**, Ch. 2.

[6] W. Williams, T. M. Wright, *J. Geophys. Res.* **1998**, *103*, 30537.

[7] K. E. Mooney, J. A. Nelson, M. J. Wagner, *Chem. Mater.* **2004**, *16*, 3155.

[8] K. J. Bishop, C. E. Wilmer, S. Soh, B. A. Grzybowski, *Small* **2009**, *5*, 1600.

[9] A. R. Muxworthy, E. McClelland, *Geophys. J. Int.* **2000**, *140*, 101.

[10] E. Lima, A. L. Brandl, A. D. Arelaro, G. F. Goya, *J. Appl. Phys.* **2006**, *99*, 083908.

[11] A. J. Rondinone, A. C. S. Samia, Z. J. Zhang, *Appl. Phys. Lett.* **2000**, *76*, 3624.

[12] N. Ranvah, Y. Melikhov, D. C. Jiles, J. E. Snyder, A. J. Moses, P. I. Williams, S. H. Song, *J. Appl. Phys.* **2008**, *103*, 07E506.

[13] A. Franco, F. L. A. Machado, V. S. Zapf, *J. Appl. Phys.* **2011**, *110*, 053913.

[14] A. López-Ortega, E. Lottini, C. d. J. Fernández, C. Sangregorio, *Chem. Mater.* **2015**, *27*, 4048.

[15] M. Sánchez-Domínguez, C. Rodríguez-Abreu, in *Nanocolloids: A Meeting Point for Scientists and Technologists* (Ed: C. Rodríguez-Abreu), Elsevier, Amsterdam **2016**, Ch. 1.

[16] M. B. Fernández van Raap, P. Mendoza Zélis, D. F. Coral, T. E. Torres, C. Marquina, G. F. Goya, F. H. Sánchez, *J. Nanopart. Res.* **2012**, *14*, 1072.

[17] G. Nahrwold, J. M. Scholtyssek, S. Motl-Ziegler, O. Albrecht, U. Merkt, G. Meier, *J. Appl. Phys.* **2010**, *108*, 013907.

[18] L. F. Yin, D. H. Wei, N. Lei, L. H. Zhou, C. S. Tian, G. S. Dong, X. F. Jin, L. P. Guo, Q. J. Jia, R. Q. Wu, *Phys. Rev. Lett.* **2006**, *97*, 067203.

[19] M. P. Ansell, in *Wood Composites* (Ed: M. P. Ansell), Woodhead Publishing, Cambridge, U. K. **2015**, Ch. 1.
